# Alkene hydroboration with pinacolborane catalysed by lithium diisobutyl-*tert*-butoxyaluminum hydride†

**DOI:** 10.1039/c9ra04699b

**Published:** 2019-08-23

**Authors:** Ashok Kumar Jaladi, Won Kyu Shin, Duk Keun An

**Affiliations:** Department of Chemistry, Institute for Molecular Science and Fusion Technology, Kangwon National University Chunchon 24341 Republic of Korea dkan@kangwon.ac.kr

## Abstract

Here we developed a highly efficient alkene hydroboration protocol, showing that various alkyl boronates can be smoothly obtained in good yields by reacting alkenes with pinacolborane (HBpin) in the presence of 5 mol% lithium diisobutyl-*tert*-butoxyaluminum hydride. The coordination of aluminate ions with lithium cations allowed for effective hydride transfer during hydroboration, and the obtained boronate ester was further used for C–C coupling, trifluoroboronate salt formation, and oxidation to alcohol.

## Abstract 2

Here we developed a highly efficient alkene hydroboration protocol, showing that various alkyl boronates can be smoothly obtained in good yields by reacting alkenes with pinacolborane (HBpin) in the presence of 5 mol% lithium diisobutyl-*tert*-butoxyaluminum hydride.

## Graphical abstract

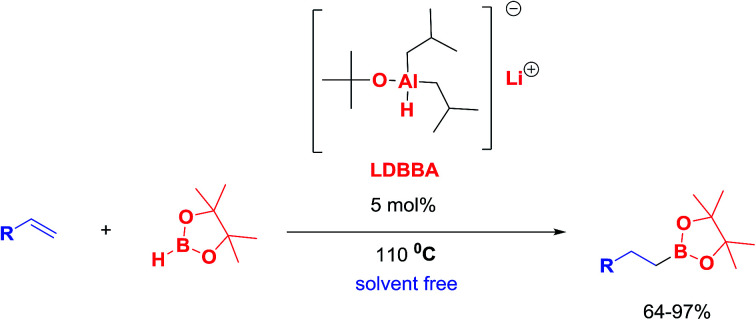

## Introduction

**Fig. 1:**
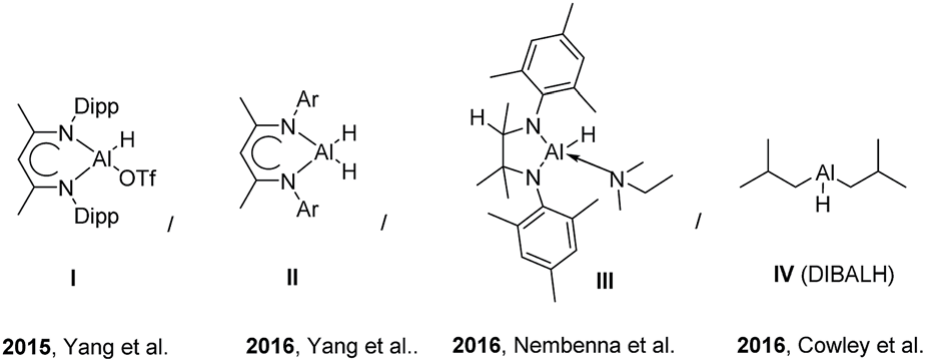
Reported aluminum hydrides for catalytic hydroboration of C=O, C=C bonds.

The increasing interest in catalytic hydro-functionalization (hydrosilylation/boration) and manipulation has inspired the development of suitable protocols for the synthesis of organometallics.^1,2^ In particular, much attention has been directed at the catalytic hydroboration of unsaturated hydrocarbons, *e.g.*, noble metal-catalyzed hydroboration of alkenes and alkynes, as these reactions afford organoboron compounds as versatile building blocks for a variety of transformations and cross-coupling reactions. In this regard, metal catalyzed (precious metal catalyst) hydroboration of alkenes and alkynes have been studied extensively to synthesize these valuable precursors.^3,4^ Although Earth-abundant transition metal and main group metal catalysts are also used in hydroboration reactions (especially in those of carbonyl compounds) and can exhibit activities similar to those of their noble metal counterparts, most of these catalysts however, correspond to pincer or phosphine ligand-containing complexes.^5^ The catalytic hydroboration of carbonyl compounds has been widely investigated, whereas that of alkenes and alkynes with readily available main group hydride reagent has received little attention^6^ (Fig. 1).

Group 13 hydrides have been extensively studied because of their ability to store hydrogen, participate in various organic transformations, and mediate the reduction of unsaturated substrates.^7^ In particular, mono- and dihydrides of Al, the third most abundant element (8.1%) in the Earth's crust [after O (46.6%) and Si (27.7%)], have been used for the hydroboration of carbonyls and alkynes.

**Scheme 1:**
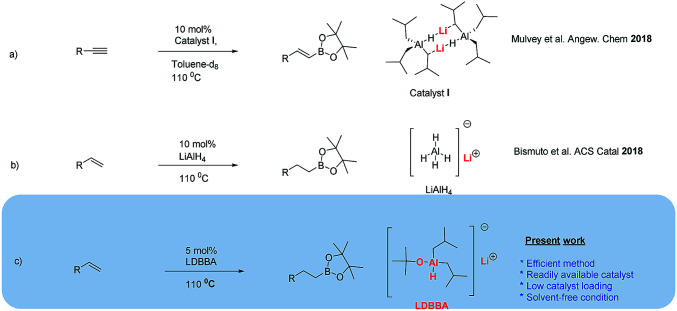
(a and b) Previously reported Al hydride-catalyzed hydroborations and (c) Al hydride-catalyzed hydroboration developed herein.

In 2015, Yang *et al.*^8^ reported [LAlH(OTf)] (L = HC(CMeNAr)_2_, Ar = 2,6-^i^Pr_2_C_6_H_3_)-catalyzed hydroboration of carbonyl groups and the addition of trimethylsilyl cyanide to aldehydes and ketones. In another investigation, Yang *et al.* reported Al dihydride LAlH_2_ (L = HC(CMeNAr)_2_, Ar = 2,6-Et_2_C_6_H_3_)-catalyzed hydroboration of terminal alkynes and dehydrocoupling of boranes with amines, phenols, and thiols in deuterated solvents.^9^ More recently, Bismuto *et al.* reported DIBAL-H (10 mol%) catalyzed hydroboration of alkynes and the hydroboration of alkenes with pinacolborane (HBpin) catalyzed by commercially available Al hydrides such as the highly reactive LiAlH_4_ and/or Na bis(2-methoxyethoxy)aluminum hydride (Red-Al), exploring the substrate scope for 10 mol% LiAlH_4_ (Scheme 1).^10,11^ Mulvey *et al.* reported that Al-containing anionic ate complexes with alkali metal (Li–Al cooperativity) featured synergistic reactivity for effective hydroboration. In their study, Mulvey *et al.* compared the hydroboration of aldehydes, ketones, imines and alkynes in the presence of bimetallic lithium aluminates and neutral aluminum counterparts as catalysts.^12^

Based on the works of Yang, Bismuto, Thomas, Mulvey, and our recent reports on selective and partial reductions promoted by Al-containing catalysts, we herein present the results of our research on the catalytic hydroboration of alkenes with lithium diisobutyl-*tert*-butoxyaluminum hydride (LDBBA) and HBpin.

Lithium diisobutyl-*tert*-butoxyaluminum hydride (LDBBA) is a new class of reducing agent know for the partial reduction of esters and amides (tertiary and Weinreb amides) to aldehydes.^13^ LDBBA was also reported for the one pot synthesis of secondary alcohols such as, vinyl and propargyl alcohols from the ester precursor.^14^ Recently, LDBBA was successfully applied in the flow chemistry to achieve the selective reduction of esters.^15^

Due to the mild nature, easy to handle and simple work up procedure associated with LDBBA, we applied for catalytic hydroboration of alkenes in an effective manner under solvent-free condition (Scheme 1).

Recently reported aluminium hydrides for catalytic hydroboration reactions are depicted in below figure.

## Results and discussion

When alkene (styrene) hydroboration was performed with 3.0 equiv. HBpin and 10 mol% catalyst (LDBBA) at room temperature, the corresponding boronate was obtained only in small yield (entry 1, Table 1). Higher conversions were obtained when the reaction temperature was increased and the loading of HBpin was reduced, *e.g.*, quantitative conversion was achieved with a 10 mol% catalyst loading within 2 h (entry 2). Based on this observation, we tried to further improve the reaction conditions. The reaction was repeated under the conditions of entry 2 with reduced catalyst loading of 5 mol%, which, again, resulted in quantitative hydroboration within 2 h (entry 3). Notably, our method proved to be more effective than the recently reported method using highly reactive LiAlH_4_.

**Table 1:** Optimization of reaction conditions for LDBBA-catalyzed hydroboration of alkenes

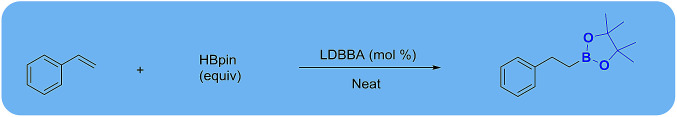					
Entry	Catalyst (mol%)	HBpin (equiv.)	Temp.	Time (h)	Conversion^a^ (%)
1	LDBBA (10)	3.0	rt	24	18%
2	LDBBA (10)	1.5	110 °C	2	99%
3	LDBBA (5)	1.5	110 °C	2	99%
**4**	**LDBBA (5)**	**1.2**	**110 °C**	**2**	**99% (91)** b
5	LDBBA (5)	1.2	110 °C	1	74%
6^c^	LDBBA (5)	1.2	110 °C	2	72%
7	LDBBA (1)	1.2	110 °C	2	57%
8	No catalyst	2.0	110 °C	12	23%
9	DIBALH (5)	1.2	110 °C	2	59%
10	LTBA (5)	1.2	110 °C	2	79%
11	Red-Al (5)	1.2	110 °C	4	85% ^11^

a Conversions were determined from GC peak area ratios based on starting material consumption.

b Isolated yield.

c Reaction with mL toluene; LTBA (lithium tri-*tert*-butoxy aluminum hydride).

Considering this results, we next decreased HBpin loading from 1.5 to 1.2 equiv., demonstrating that quantitative hydroboration was achieved. The resulting crude boronate was extracted with ethyl acetate, volatiles evaporation followed by column chromatography from silica gel affording the isolated yield of the corresponding alkyl boronate equaled 91% (entry 4). However, conversion decreased to 74% when the reaction time was reduced to 1 h (entry 5). Although alkene hydroboration was also observed for catalyst loading of 1 and 0 mol% at higher HBpin loadings, no significant conversion to product was achieved in both cases (entries 6 and 7). Therefore, it was concluded that optimal conditions for the conversion of alkene to alkyl boronate correspond to 5 mol% LDBBA, 1.2 equiv. HBpin, 110 °C, and a reaction time of 2 h (entry 4).

**Scheme 2:**
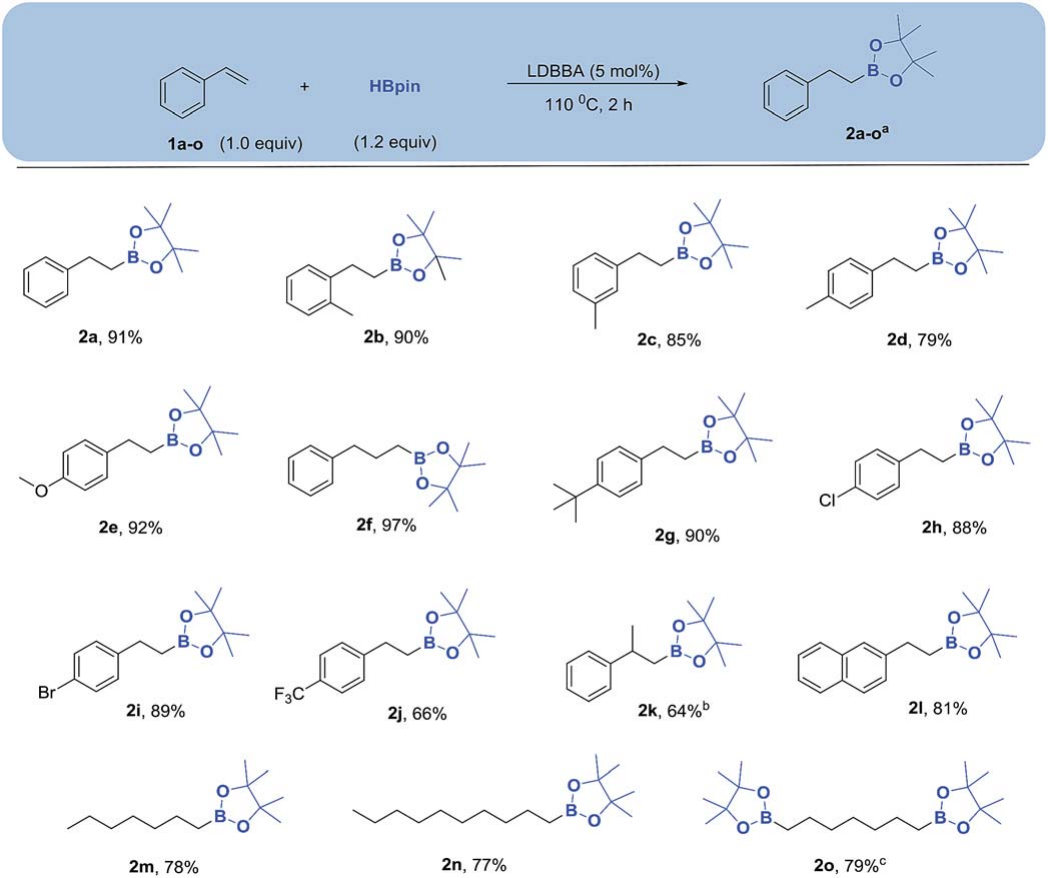
Substrate scope for LDBBA-catalyzed hydroboration of alkenes. Reaction condition: alkene (1.0 mmol), HBpin (1.2 mmol), LDBBA (5 mol%), 110 °C, 2 h; ^*a*^isolated yields; ^*b*^HBpin (2.0 equiv.), 6 h; ^*c*^HBpin (2.4 equiv.), 4 h.

With the optimized conditions in hand, we explored the substrate scope (Scheme 2), revealing that both electron-rich alkenes (bearing 2-methyl, 3-methyl, 4-methyl, 4-methoxy, 4-*tert*-butyl substituents) and electron-poor alkenes (bearing 4-chloro, 4-bromo and 4-fluoro substituents) reacted in the same manner to afford the corresponding boronates in good yields. Allylbenzene was converted to the expected product in good yield (97%), while a longer reaction time was required for an alpha methyl-substituted styrene (prop-1-en-2-ylbenzene). A polyaromatic substrate 2-vinylnaphthalene and an aliphatic alkenes (1-heptene, 1-decene) were converted to the corresponding boronate esters in good yields. In addition, a dialkene 1,7-heptene was also afforded corresponding bis-boronate smoothly in good yield.

**Scheme 3:**
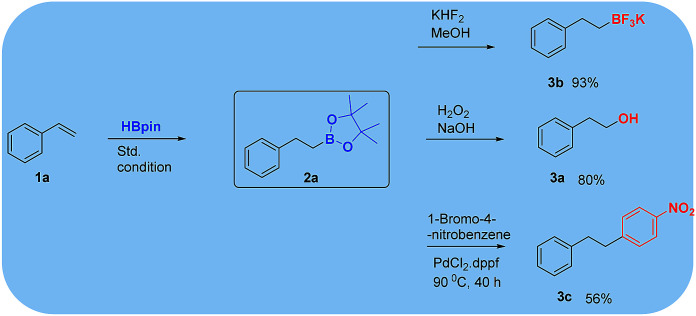
Transformation of 4,4,5,5-tetramethyl-2-phenethyl-1,3,2-dioxaborolane 2a.

Because of their significant utility, boronates are often transformed into other substances in organic synthesis. Herein, boronate ester 2a was treated with H_2_O_2_ in the presence of aqueous NaOH or methanolic KHF_2_ to afford the corresponding alcohol or trifluoro boronate products, respectively, in good yield.^16^ Further, the Suzuki reaction of 2a with 4-nitrobromobenzene afforded 1-nitro-4-phenethylbenzene (Scheme 3).^17^

## Conclusions

In conclusion, we demonstrated that alkenes can be efficiently hydroborated in the presence of commercial lithium diisobutyl-*tert*-butoxyaluminum hydride (LDBBA), revealing that a catalyst loading of 5 mol% is sufficient to produce alkyl boronates from a variety of substituted alkenes in good to excellent yields. In addition, boronate 2a was used in a cross-coupling reaction and employed to prepare synthetically valuable synthons such as the corresponding alcohol and potassium trifluoroborate salt. The coordination of anionic aluminate with lithium allowed for effective hydride transfer during hydroboration. Thus, the present method of alkene hydroboration is a good alternative to the existing complex transition and precious metal-mediated hydroborations.

## Conflicts of interest

There are no conflicts to declare.

## Supplementary Material

RA-009-C9RA04699B-s001

## References

[cit1] Synthesis and application of organoboron compounds, ed. E. Fernandez and A. Whiting, Springer, Heidelberg, 2015

[cit2] Brown H. C., Rao B. C. S. (1956). J. Am. Chem. Soc..

[cit3] Mannig D., Nöth H. (1985). Angew. Chem., Int. Ed. Engl..

[cit4] Miyaura N., Yamada K., Suzuki A. (1979). Tetrahedron Lett..

[cit5] Zhang H., Lu Z. (2016). ACS Catal..

[cit6] Jakhar V. K., Barman M. K., Nembenna S. (2016). Org. Lett..

[cit7] Downs A. J., Pulham C. R. (1994). Chem. Soc. Rev..

[cit8] Yang Z., Zhong M., Ma X., De S., Anusha C., Parameswaran P., Roesky H. W. (2015). Angew. Chem., Int. Ed..

[cit9] Yang Z., Zhong M., Ma X., Nijesh K., De S., Parameswaran P., Roesky H. W. (2016). J. Am. Chem. Soc..

[cit10] Bismuto A., Thomas S. P., Cowley M. J. (2016). Angew. Chem., Int. Ed..

[cit11] Bismuto A., Cowley M. J., Thomas S. P. (2018). ACS Catal..

[cit12] Pollard V. A., Fuentes M. A., Kennedy A. R., McLellan R., Mulvey R. E. (2018). Angew. Chem., Int. Ed..

[cit13] Kim M. S., Mi Y., An D. K. (2007). Tetrahedron Lett..

[cit14] Chae M. J., Jeon A. R., Livinghouse T., An D. K. (2011). Chem. Commun..

[cit15] Muñoz J. M., Alcázar J., Hoz A., Díaz-Ortiz A. (2012). Eur. J. Org. Chem..

[cit16] Yanga K., Song Q. (2016). Green Chem..

[cit17] Molander G. A., Yun C. S., Ribagorda M., Biolatto B. (2003). J. Org. Chem..

