# Supplementary material for: Alkene hydroboration with pinacolborane catalysed by lithium diisobutyl-tert-butoxyaluminum hydride
Source: RSC Adv. 2019 Aug 23;9(45):26483–6. doi: 10.1039/c9ra04699b (PMC9070384; doi:10.1039/c9ra04699b)
Supplement: RA-009-C9RA04699B-s001 [file RA-009-C9RA04699B-s001.pdf]

Electronic Supplementary Material (ESI) for RSC Advances.
This journal is © The Royal Society of Chemistry 2019

Supporting Information

Alkene hydroboration with pinacolborane catalysed by lithium diisobutyl-*tert*-butoxyaluminum hydride

Ashok Kumar Jaladi, Won Kyu Shin and Duk Keun An*

Department of Chemistry, Kangwon National University

Chuncheon 24341, Republic of Korea

E-mail: dkan@kangwon.ac.kr

Table of Contents

1. Experimental S1

General Information S1

General procedure for catalyzed hydroboration of alkenes S1

Procedure for the oxidation of boronate to 2-phenylethan-1-ol S1

Procedure for the synthesis of potassium phenethyltrifluoroborate S2

Procedure for the synthesis of 1-nitro-4-phenethylbenzene S2

2. Characterization of the products S3-S6

3. Copies of NMR spectra S7-S23

4. References S24

1. Experimental section:

General Information:

All glassware used was dried thoroughly in an oven, assembled hot, and cooled under a stream of dry nitrogen prior to use. All chemicals were commercial products of the highest purity. HBpin and alkenes were purchased from Aldrich Chemical Company. ^{1}H NMR spectra were measured at 400 MHz with $CDCl_3$ as a solvent at ambient temperature unless otherwise indicated and the chemical shifts were recorded in parts per million downfield from tetramethylsilane (δ = 0 ppm) or based on residual $CDCl_3$ (δ = 7.26 ppm) as the internal standard. ^{13}C NMR spectra were recorded at 100 MHz with $CDCl_3$ as a solvent and referenced to the central line of the solvent (δ = 77.0 ppm). The coupling constants (*J*) are reported in hertz. Analytical thin-layer chromatography (TLC) was performed on glass precoated with silica gel (Merck, silica gel 60 F254). Column chromatography was carried out using 70-230 mesh silica gel (Merck) at normal pressure. GC analyses were performed on a Younglin Acme 6100M and 6500 GC FID chromatography, using an HP-5 capillary column (30 m). All GC yields were determined with the use of naphthalene as the internal standard and the authentic sample.

General procedures for the LDBBA catalyzed hydroboration of alkenes (2a-l):

A 20 mL test tube was charged with styrene (1.0 mmol, 0.11 mL), pinacolborane (1.2 mmol, 0.18 mL) at room temperature. To this LDBBA (5 mol%, 0.39 M, 0.13 mL) was added under nitrogen atmosphere at the same temperature, reaction mixture was brought to 110 °C and stirred for 2 h. After this time, reaction mixture was cooled to room temperature, unreacted substrates were quenched by the addition of 1 mL of water. The crude mixture was extracted with ethyl acetate and combined organic layers were dried over $MgSO_4$. (Conversions were determined by Gas chromatography). Solvents (volatiles) were evaporated under reduced pressure, residue mixture was subjected to column chromatography using silica gel. Isolated compounds were analyzed by spectroscopic data, compared with literature.

Procedure for the oxidation of boronate to 2-phenylethan-1-ol (3a)[S1]

The substrate 4,4,5,5-Tetramethyl-2-phenethyl-1,3,2-dioxaborolane (**2a**, 1 mmol, 232 mg) was taken in 25 mL round bottom flask and dissolved in 2 mL of THF. To this NaOH (5 eq, 5 mmol, 200 mg) was added at 0 °C, followed by 30% H_2O_2 (5 eq, 0.5 mL) was added and stirring was continued for 1 h at the same temperature. After completion of the reaction, H_2O (4 mL), EtOAC (4 mL) were added and decanted, filtered through $MgSO_4$. The crude mixture was isolated under short column chromatography with ethyl acetate:hexane to afford the pure substance as a colorless liquid in 80% yield.

Procedure for the synthesis of potassium phenethyltrifluoroborate (3b)[S1,S2]

The substrate 4,4,5,5-Tetramethyl-2-phenethyl-1,3,2-dioxaborolane (**2a**, 1 mmol, 232 mg) was taken in 25 mL round bottom flask and dissolved in 5 mL methanol. To this, KHF_2 (1 mL, 4.5 M) was added drop wise at room temperature, reaction mixture was stirred for 30 min. After this time solvent was evaporated under reduced pressure. To this crude material, hot acetone was added (3x10 mL), decanted. Filtrate (decanted liquid) was evaporated to leave a white solid. To this solid, ether was and decanted to remove pinacol and dried under vaccume to obtain pure potassium phenethyltrifluoroborate as colorless solid in 93% yield.

Procedure for the synthesis of 1-nitro-4-phenethylbenzene (3c)[S3]

A 25 mL round bottom flask was charged with **2a** (0.5 mmol, 116 mg), 1-bromo-4-nitrobenzene (2 eq, 1.0 mmol, 202 mg), Cs_2CO_3 (3 eq, 1.5 mmol, 487 mg) in 5 mL THF, 0.5 mL H_2O. To this $PdCl_2$dppf (5 mol%, 20 mg), was added, and purged with argon. The reaction mixture was brought to 90 °C and stirred for 40 h. After this time, solvent was evaporated and extracted with ethyl acetate. The combined organic layers were dried over $MgSO_4$, solvent was evaporated under reduced pressure. The crude material was purified under column chromatography using silica gel with ethyl acetate and hexane to afford 1-nitro-4-phenethylbenzene (**3c**) as a pale yellow solid in 56 % yield.

2. Characterization of the products

4,4,5,5-Tetramethyl-2-phenethyl-1,3,2-dioxaborolane (2a)[S4]:

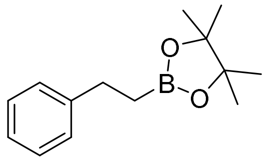

According to general procedure, compound **2a** was prepared in 91% (0.91 mmol, 211 mg) yield as colorless liquid. ^{1}H NMR (400 MHz, $CDCl_3$) δ 7.29 – 7.19 (m, 4H), 7.18 – 7.12 (m, 1H), 2.79 – 2.70 (m, 2H), 1.22 (s, 12H, CH_2x4), 1.18 – 1.12 (m, 2H). ^{13}C NMR (100 MHz, $CDCl_3$) δ 144.52, 128.33, 128.13, 125.65, 83.23, 30.08, 24.93.

4,4,5,5-Tetramethyl-2-(2-methylphenethyl)-1,3,2-dioxaborolane (2b)[S5]:

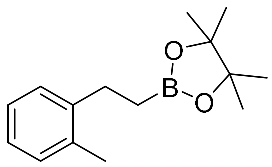

According to general procedure, compound **2b** was prepared in 90% (0.90 mmol, 221 mg) yield as colorless liquid. ^{1}H NMR (400 MHz, $CDCl_3$) δ 7.21 – 7.02 (m, 4H), 2.71 (t, J = 8.3 Hz, 2H), 2.31 (s, 3H), 1.23 (s, 12H), 1.09 (t, J = 8.1 Hz, 2H). ^{13}C NMR (100 MHz, $CDCl_3$) δ 142.62, 135.90, 130.05, 128.16, 125.95, 125.72, 83.21, 27.27, 24.93, 19.41.

4,4,5,5-Tetramethyl-2-(3-methylphenethyl)-1,3,2-dioxaborolane (2c)[S5]:

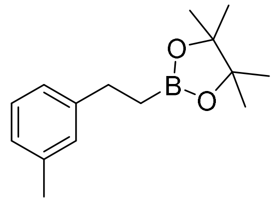

According to general procedure, compound **2c** was prepared in 85% (0.85 mmol, 209 mg) yield as colorless liquid.^{1}H NMR (400 MHz, $CDCl_3$) δ 7.14 (t, J = 7.5 Hz, 1H), 7.07 – 6.93 (m, 3H), 2.75 – 2.64 (m, 2H), 2.31 (s, 3H), 1.22 (s, 12H), 1.16 – 1.08 (m, 1H). ^{13}C NMR (100 MHz, $CDCl_3$) δ 144.49, 137.75, 128.96, 128.21, 126.33, 125.11, 83.18, 29.97, 24.91, 21.52.

4,4,5,5-Tetramethyl-2-(4-methylphenethyl)-1,3,2-dioxaborolane (2d)[S5]:

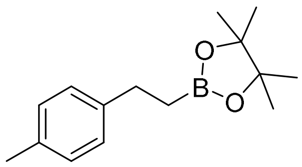

According to general procedure, compound **2d** was prepared in 79% (0.79 mmol, 194 mg) yield as colorless liquid.^{1}H NMR (400 MHz, $CDCl_3$) δ 7.08 (q, J = 8.0 Hz, 4H), 2.74 – 2.64 (m, 2H), 2.30 (s, 3H), 1.22 (s, 12H), 1.14 – 1.08 (m, 2H). ^{13}C NMR (100 MHz, $CDCl_3$) δ 141.50, 134.97, 128.98, 127.94, 83.18, 29.61, 24.92, 21.09.

2-(4-Methoxyphenethyl)-4,4,5,5-tetramethyl-1,3,2-dioxaborolane (2e)[S4]:

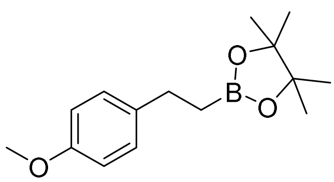

According to general procedure, compound **2e** was prepared in 92% (0.92 mmol, 241 mg) yield as colorless liquid.^{1}H NMR (400 MHz, $CDCl_3$) δ 7.12 (d, J = 8.6 Hz, 2H), 6.80 (d, J = 8.7 Hz, 2H), 3.77 (s, 3H, OCH_3), 2.72 – 2.64 (m, 2H), 1.21 (s, 12H), 1.14 – 1.07 (m, 2H). ^{13}C NMR (100 MHz, $CDCl_3$) δ 157.62, 136.66, 128.97, 113.67, 83.17, 55.35, 29.14, 24.91.

4,4,5,5-Tetramethyl-2-(3-phenylpropyl)-1,3,2-dioxaborolane (2f)[S4]:

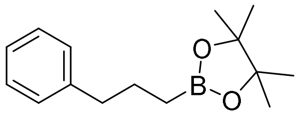

According to general procedure, compound **2f** was prepared in 97% (0.97 mmol, 238 mg) yield as colorless liquid.^{1}H NMR (400 MHz, $CDCl_3$) δ 7.29 – 7.21 (m, 2H), 7.19 – 7.11 (m, 3H), 2.64 – 2.54 (m, 2H), 1.80 – 1.64 (m, 2H), 1.23 (s, 12H), 0.82 (t, J = 7.9 Hz, 2H). ^{13}C NMR (100 MHz, $CDCl_3$) δ 142.80, 128.66, 128.28, 125.67, 83.04, 38.71, 26.24, 24.93.

2-(4-(Tert-butyl)phenethyl)-4,4,5,5-tetramethyl-1,3,2-dioxaborolane (2g)[S4]:

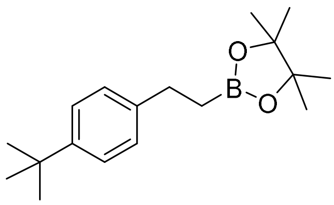

According to general procedure, compound **2g** was prepared in 90% (0.90 mmol, 260 mg) yield as colorless liquid.^{1}H NMR (400 MHz, $CDCl_3$) δ 7.28 (d, J = 8.3 Hz, 2H), 7.15 (d, J = 8.3 Hz, 2H), 2.75 – 2.67 (m, 2H), 1.29 (s, 9H), 1.22 (s, 12H), 1.16 – 1.10 (m, 2H). ^{13}C NMR (100 MHz, $CDCl_3$) δ 148.33, 141.46, 127.73, 125.18, 83.17, 34.41, 31.53, 29.47, 24.91.

2-(4-Chlorophenethyl)-4,4,5,5-tetramethyl-1,3,2-dioxaborolane (2h)[S6]:

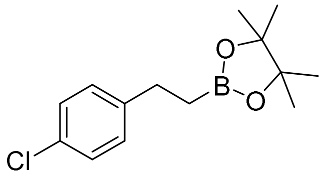

According to general procedure, compound **2h** was prepared in 88% (0.88 mmol, 234 mg) yield as colorless liquid.^{1}H NMR (400 MHz, $CDCl_3$) δ 7.20 (d, J = 8.4 Hz, 2H), 7.12 (d, J = 8.5 Hz, 2H), 2.75 – 2.65 (m, 2H), 1.20 (s, 12H), 1.13 – 1.06 (m, 2H). ^{13}C NMR (100 MHz, $CDCl_3$) δ 142.91, 131.23, 129.49, 128.33, 83.29, 29.42, 24.90.

2-(4-Bromophenethyl)-4,4,5,5-tetramethyl-1,3,2-dioxaborolane (2i)[S6]:

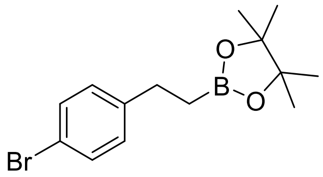

According to general procedure, compound **2i** was prepared in 89% (0.89 mmol, 277 mg) yield as colorless liquid.^{1}H NMR (400 MHz, $CDCl_3$) δ 7.35 (d, J = 8.4 Hz, 2H), 7.07 (d, J = 8.4 Hz, 2H), 2.73 – 2.63 (m, 2H), 1.20 (s, 12H), 1.12 – 1.07 (m, 2H). ^{13}C NMR (100 MHz, $CDCl_3$) δ 143.44, 131.28, 129.92, 83.29, 29.48, 24.90.

4,4,5,5-tetramethyl-2-(4-(trifluoromethyl)phenethyl)-1,3,2-dioxaborolane (2j)[S5]:

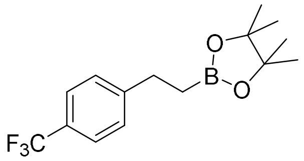

According to general procedure, compound **2j** was prepared in 66% (0.66 mmol, 198 mg) yield as colorless liquid. ^{1}H NMR (400 MHz, $CDCl_3$) δ 7.49 (d, J = 8.2 Hz, 2H), 7.30 (d, J = 8.2 Hz, 2H), 2.78 (t, J = 8.2 Hz, 1H), 1.20 (s, 12H CH_3x4), 1.16 – 1.10 (m, 2H). ^{13}C NMR (100 MHz, $CDCl_3$) δ 148.59, 128.40, 128.0 (q, J = 32.1 Hz), 125.2 (q, J = 3.8 Hz), 124.46 (q, J = 272 Hz), 83.34, 29.91, 24.86.

4,4,5,5-Tetramethyl-2-(2-phenylpropyl)-1,3,2-dioxaborolane (2k)[S4]:

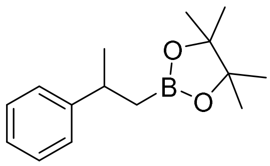

According to general procedure, compound **2k** was prepared in 64% (0.64 mmol, 157 mg) yield as colorless liquid.^{1}H NMR (400 MHz, $CDCl_3$) δ 7.32 – 7.18 (m, 4H), 7.13 (tt, *J* = 5.8, 2.2 Hz, 1H), 3.09 – 2.92 (m, 1H), 1.26 (d, *J* = 6.9 Hz, 3H), 1.15 (s, 14H). ^{13}C NMR (100 MHz, $CDCl_3$) δ 149.31, 128.27, 126.72, 125.77, 83.08, 35.90, 25.01, 24.86, 24.77.

4,4,5,5-Tetramethyl-2-(2-(naphthalen-2-yl)ethyl)-1,3,2-dioxaborolane (2l)[S5]:

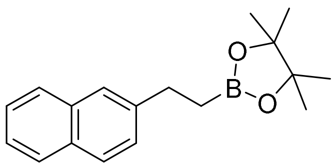

According to general procedure, compound **2l** was prepared in 81% (0.81 mmol, 228 mg) yield as colorless liquid.^{1}H NMR (400 MHz, $CDCl_3$) δ 7.81 – 7.72 (m, 3H), 7.64 (s, 1H), 7.49 – 7.32 (m, 3H), 2.96 – 2.87 (m, 2H), 1.26 – 1.22 (m, 2H), 1.22 (s, 12H). ^{13}C NMR (100 MHz, $CDCl_3$) δ 142.07, 133.71, 131.98, 127.80, 127.67, 127.53, 127.38, 125.81 (d, *J* = 5.3 Hz), 125.03, 83.25, 30.22, 24.93.

2-Heptyl-4,4,5,5-tetramethyl-1,3,2-dioxaborolane (2m)[S7]:

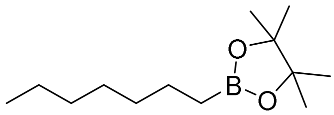

According to general procedure, compound **2m** was prepared in 78% (0.78 mmol, 176 mg) yield as colorless liquid.^{1}H NMR (400 MHz, $CDCl_3$) δ 1.38 (t, *J* = 7.4 Hz, 2H), 1.31 – 1.17 (m, 20H), 0.90 – 0.81 (m, 3H), 0.75 (t, *J* = 7.8 Hz, 2H). ^{13}C NMR (100 MHz, $CDCl_3$) δ 82.92, 32.51, 31.91, 29.20, 24.90, 24.11, 22.77, 14.22,

2-Decyl-4,4,5,5-tetramethyl-1,3,2-dioxaborolane (2n)[S7]:

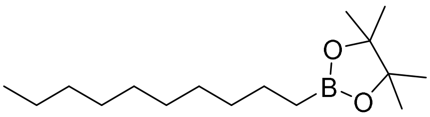

According to general procedure, compound **2n** was prepared in 77% (0.77 mmol, 206 mg) yield as colorless liquid.^{1}H NMR (400 MHz, $CDCl_3$) δ 1.43 – 1.16 (m, 28H), 0.89 – 0.82 (m, 3H), 0.75 (t, *J* = 7.8 Hz, 2H). ^{13}C NMR (100 MHz, $CDCl_3$) δ 82.91, 32.53, 32.01, 29.71, 29.68, 29.50, 29.43, 24.89, 24.09, 22.77, 14.20.

1,7-Bis(4,4,5,5-tetramethyl-1,3,2-dioxaborolan-2-yl)heptane (2o)[S8]:

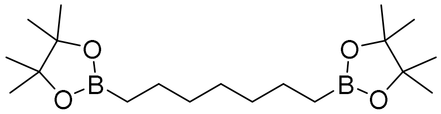

According to general procedure, compound **2o** was prepared in 79% (0.79 mmol, 268 mg) yield as colorless liquid.^{1}H NMR (400 MHz, $CDCl_3$) δ 1.40 – 1.32 (m, 4H), 1.28 – 1.16 (m, 30H), 0.73 (td, *J* = 7.8, 2.8 Hz, 4H). ^{13}C NMR (100 MHz, $CDCl_3$) δ 82.89, 32.45, 29.27, 24.89, 24.07.

2-Phenylethan-1-ol (3a)[S1]:

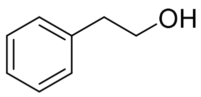

Colorless liquid. Yield. 80% (0.80 mmol, 98 mg).^{1}H NMR (400 MHz, $CDCl_3$) δ 7.36 – 7.28 (m, 2H), 7.23 (tq, *J* = 4.9, 1.7 Hz, 3H), 3.85 (t, *J* = 6.5 Hz, 2H), 2.86 (t, *J* = 6.6 Hz, 2H).

Potassium phenethyltrifluoroborate (3b)[S1,S2]:

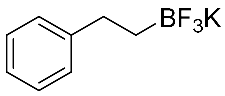

Colorless solid. Yield. 93% (0.93 mmol, 198 mg). ^{1}H NMR (400 MHz, DMSO-d6) δ 7.14 (t, *J* = 7.5 Hz, 2H), 7.10 – 7.03 (m, 2H), 7.04 – 6.97 (m, 1H), 2.41 – 2.27 (m, 2H), 0.30 – 0.12 (m, 2H).

1-Nitro-4-phenethylbenzene (3c)[S3]:

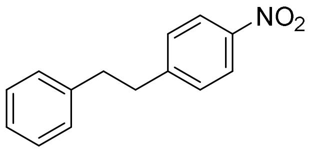

Pale yellow solid. Yield. 56% (0.28 mmol, 63 mg).^{1}H NMR (400 MHz, $CDCl_3$) δ 8.11 (d, *J* = 8.6 Hz, 2H), 7.30 – 7.23 (m, 4H), 7.23 – 7.17 (m, 1H), 7.12 (dd, *J* = 7.1, 1.8 Hz, 2H), 3.03 (ddd, *J* = 9.0, 6.7, 2.0 Hz, 2H), 2.94 (ddd, *J* = 8.5, 6.8, 2.0 Hz, 2H). ^{13}C NMR (100 MHz, $CDCl_3$) δ 149.51, 146.52, 140.56, 129.45, 128.57 (d, *J* = 7.1 Hz), 126.42, 123.70, 37.77, 37.32.

3. Copies of NMR spectra

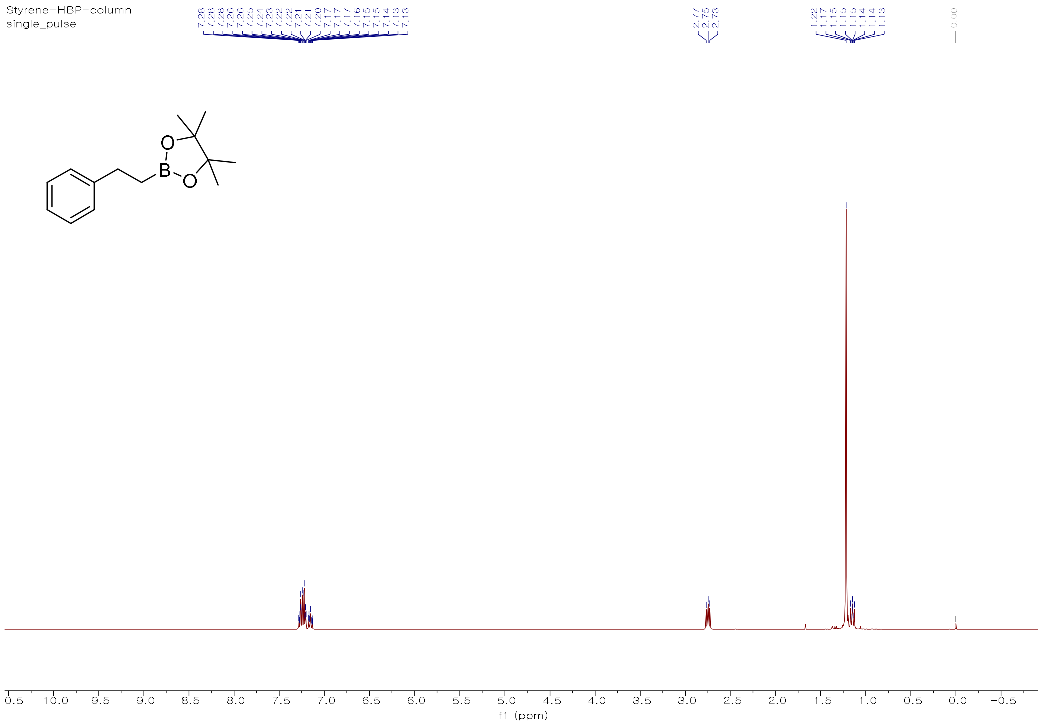

^{1}H NMR of 4,4,5,5-Tetramethyl-2-phenethyl-1,3,2-dioxaborolane (**2a**)

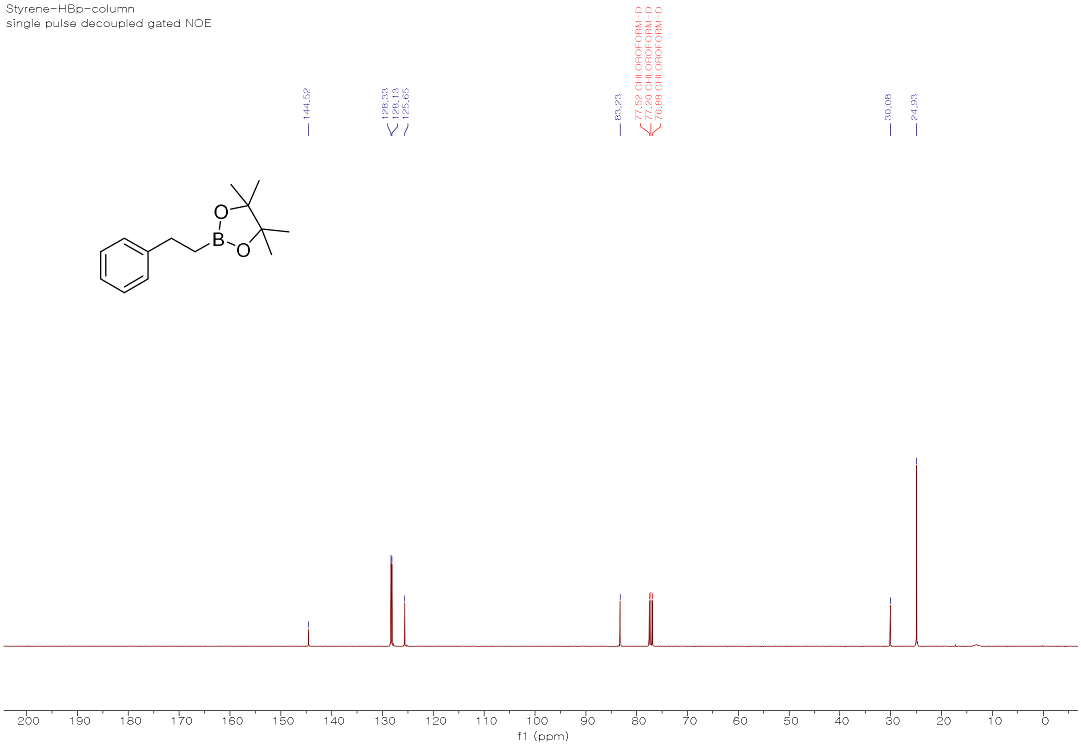

^{13}C NMR of 4,4,5,5-Tetramethyl-2-phenethyl-1,3,2-dioxaborolane (**2a**)

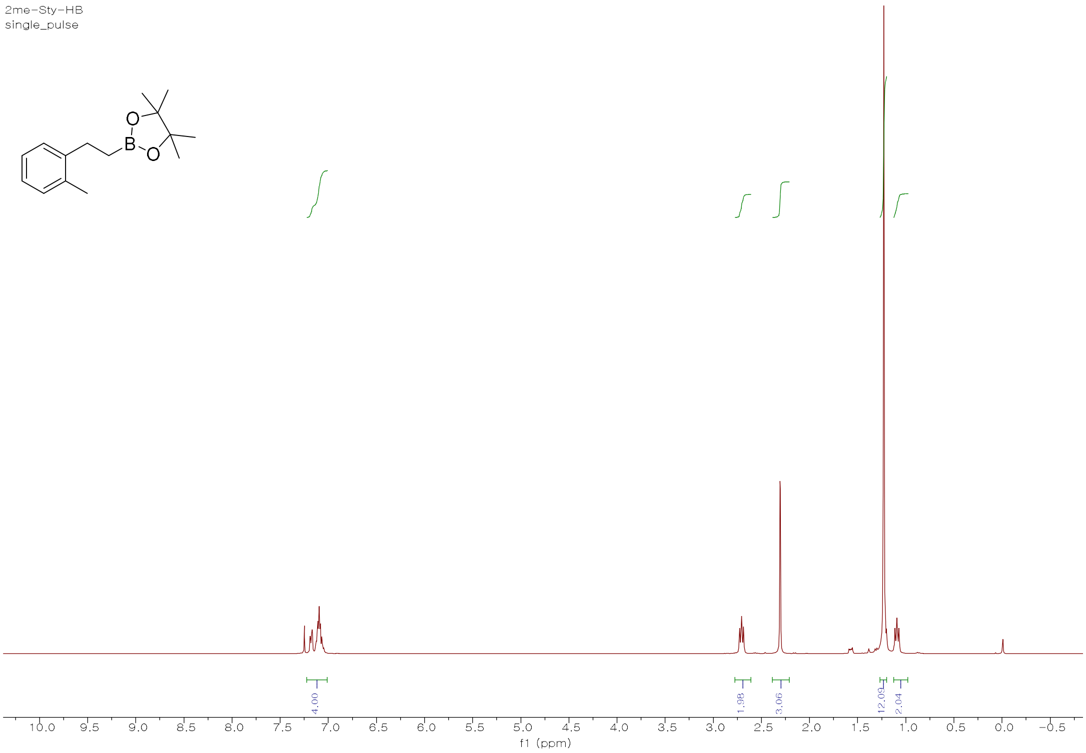

^{1}H NMR of 4,4,5,5-tetramethyl-2-(2-methylphenethyl)-1,3,2-dioxaborolane (**2b**)

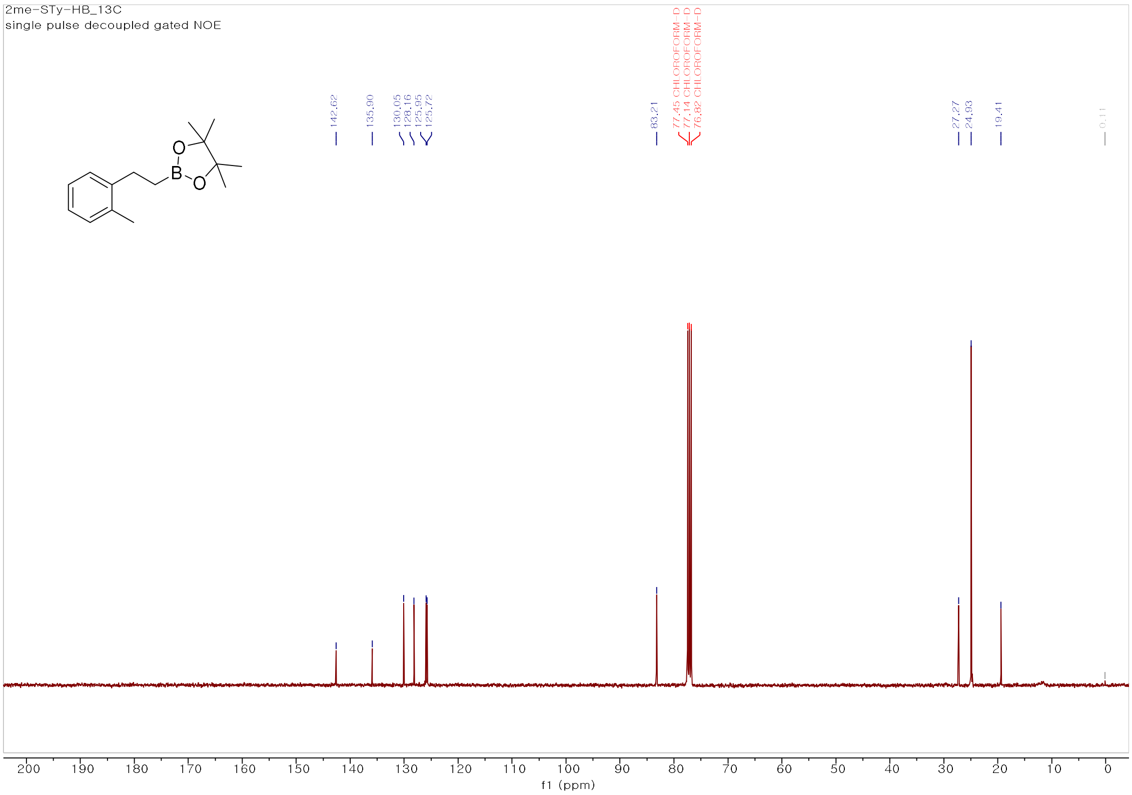

^{13}C NMR of 4,4,5,5-tetramethyl-2-(2-methylphenethyl)-1,3,2-dioxaborolane (**2b**)

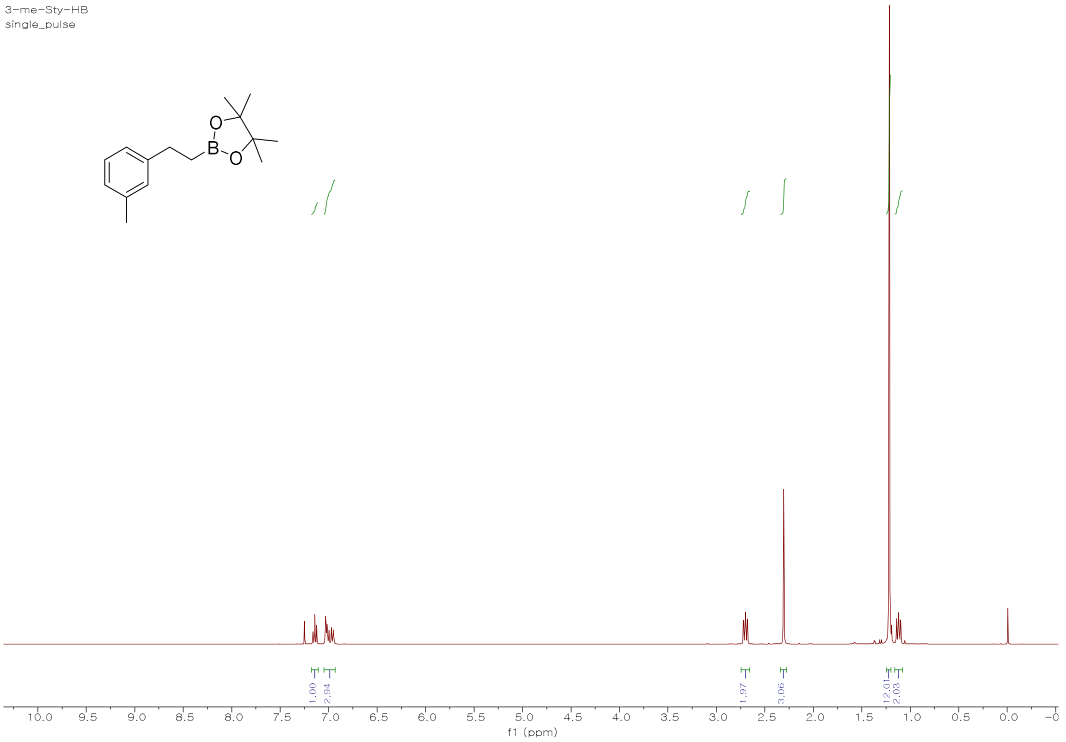

^{1}H NMR of 4,4,5,5-tetramethyl-2-(3-methylphenethyl)-1,3,2-dioxaborolane (**2c**)

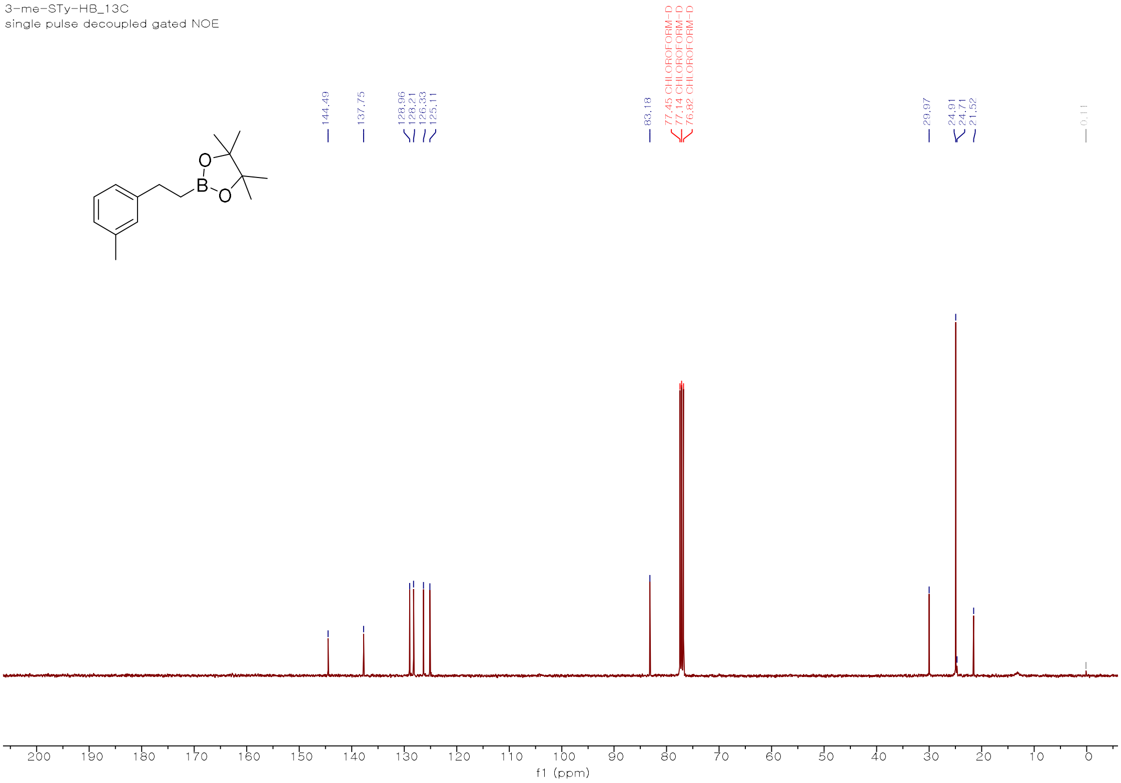

^{13}C NMR of 4,4,5,5-Tetramethyl-2-(3-methylphenethyl)-1,3,2-dioxaborolane (**2c**)

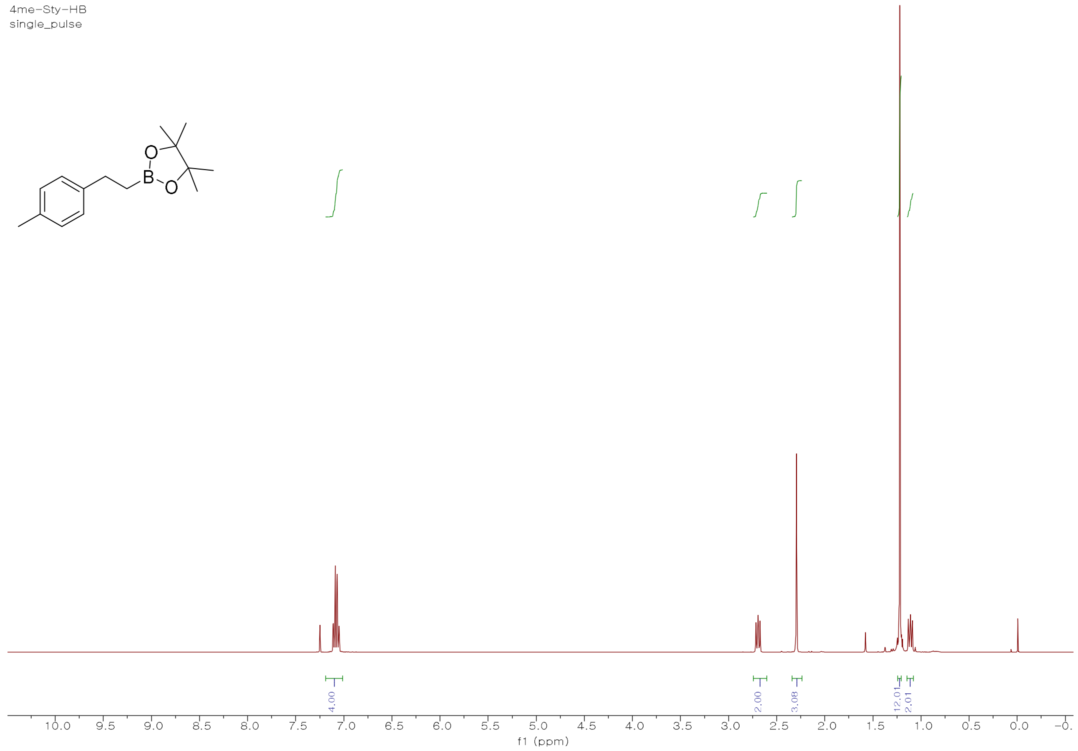

^{1}H NMR of 4,4,5,5-tetramethyl-2-(4-methylphenethyl)-1,3,2-dioxaborolane (**2d**)

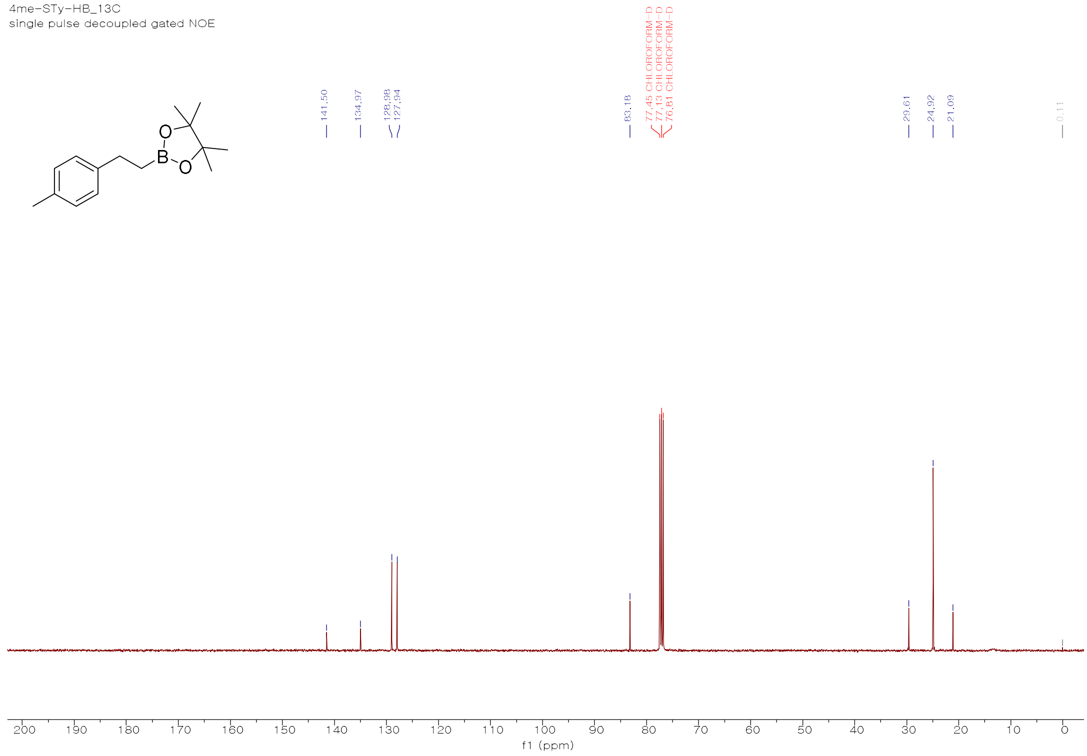

^{13}C NMR of 4,4,5,5-Tetramethyl-2-(4-methylphenethyl)-1,3,2-dioxaborolane (**2d**)

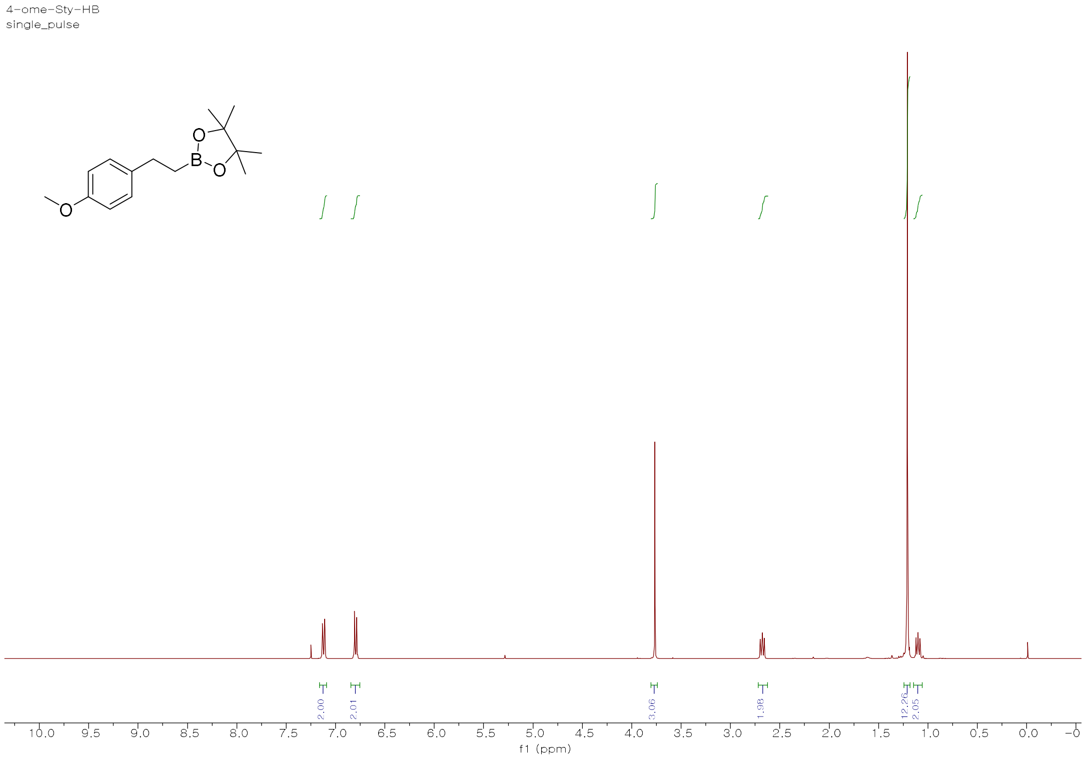

^{1}H NMR of 2-(4-methoxyphenethyl)-4,4,5,5-tetramethyl-1,3,2-dioxaborolane (**2e**)

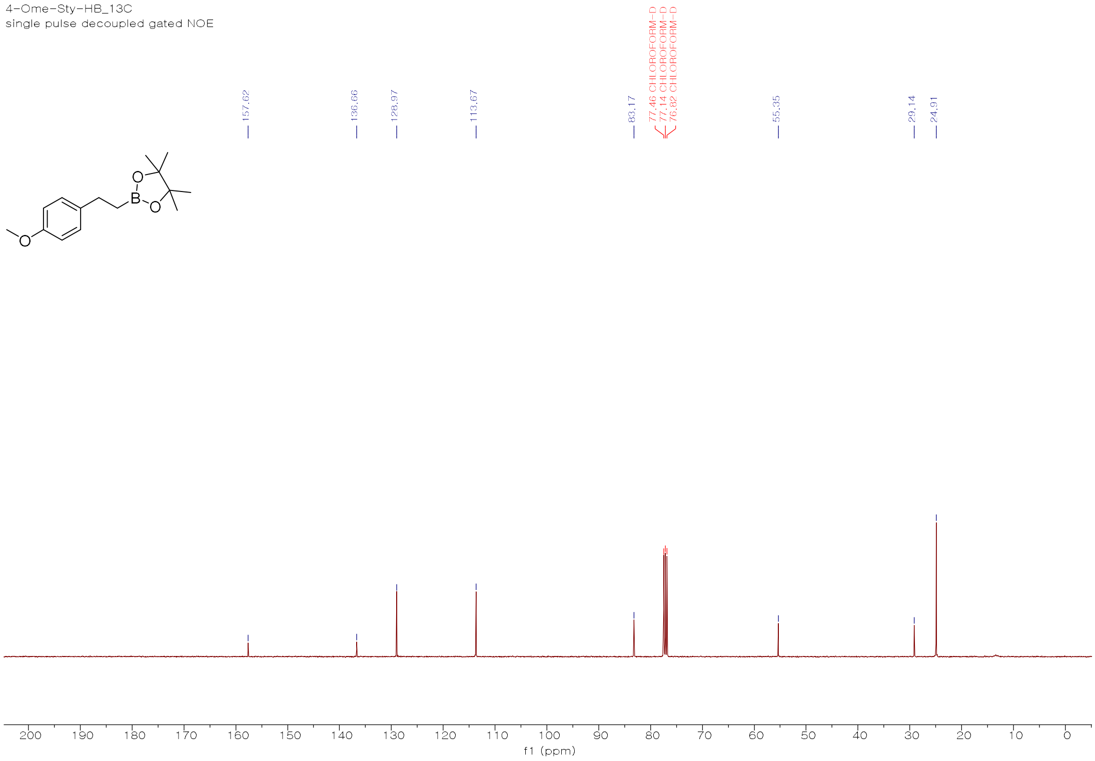

^{13}C NMR of 2-(4-methoxyphenethyl)-4,4,5,5-tetramethyl-1,3,2-dioxaborolane (**2e**)

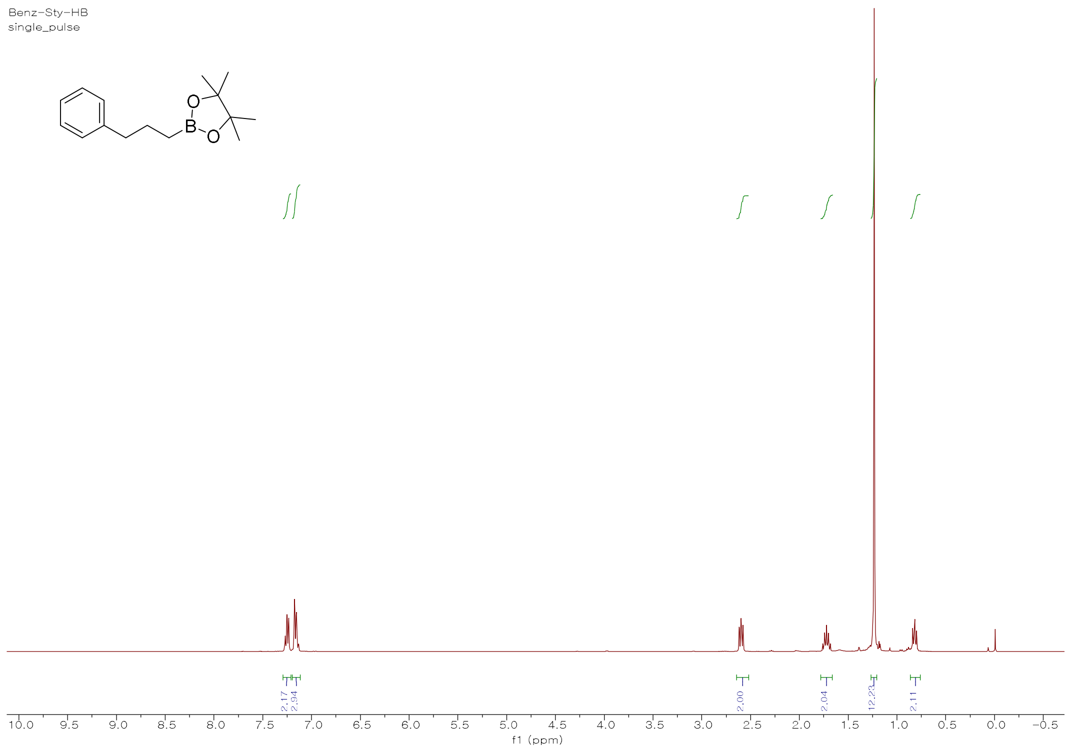

^{1}H NMR of 4,4,5,5-tetramethyl-2-(3-phenylpropyl)-1,3,2-dioxaborolane (**2f**)

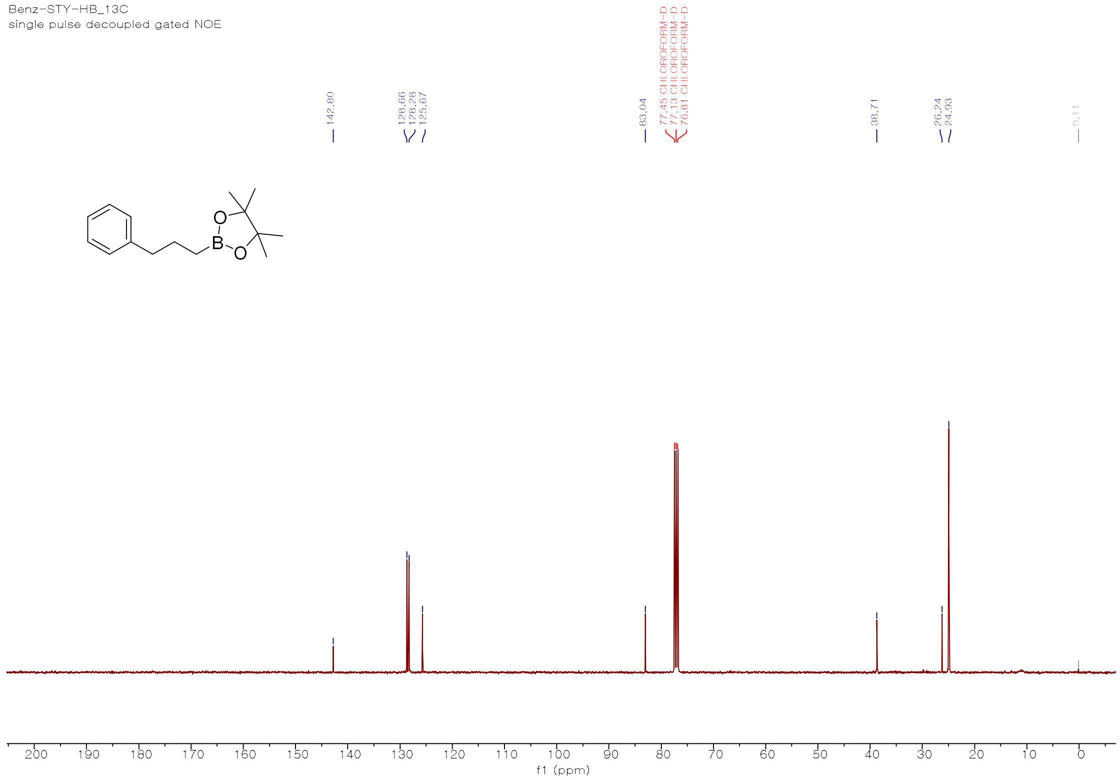

^{13}C NMR of 4,4,5,5-tetramethyl-2-(3-phenylpropyl)-1,3,2-dioxaborolane (**2f**)

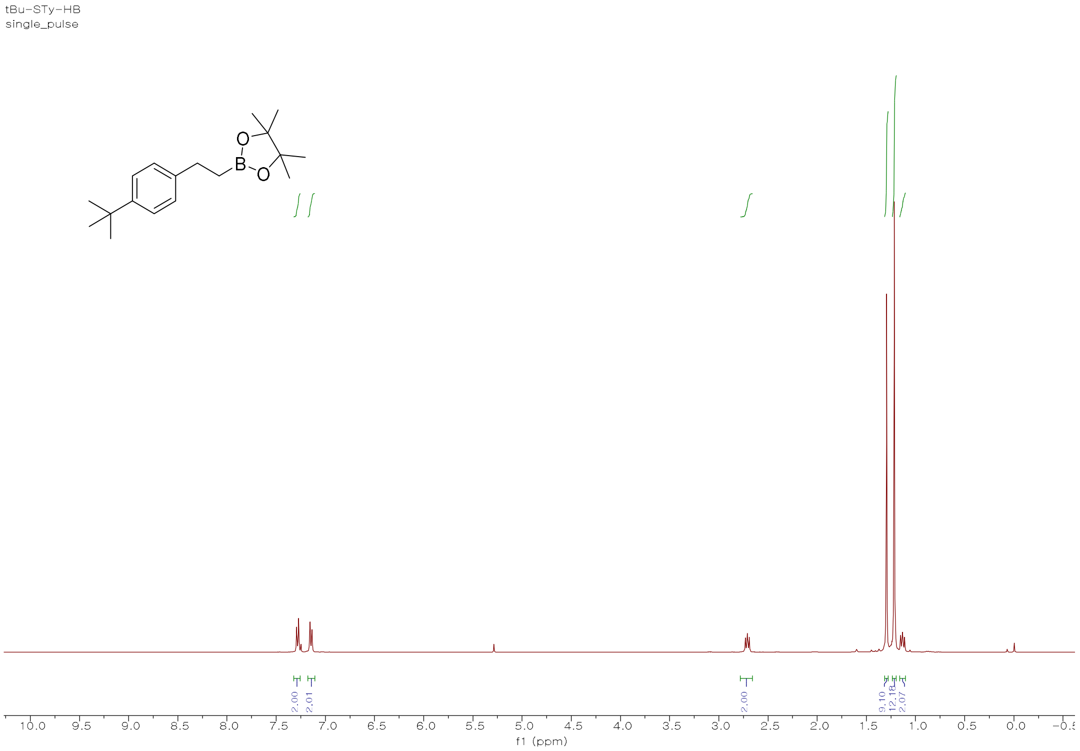

^{1}H NMR of 2-(4-(tert-butyl)phenethyl)-4,4,5,5-tetramethyl-1,3,2-dioxaborolane (**2g**)

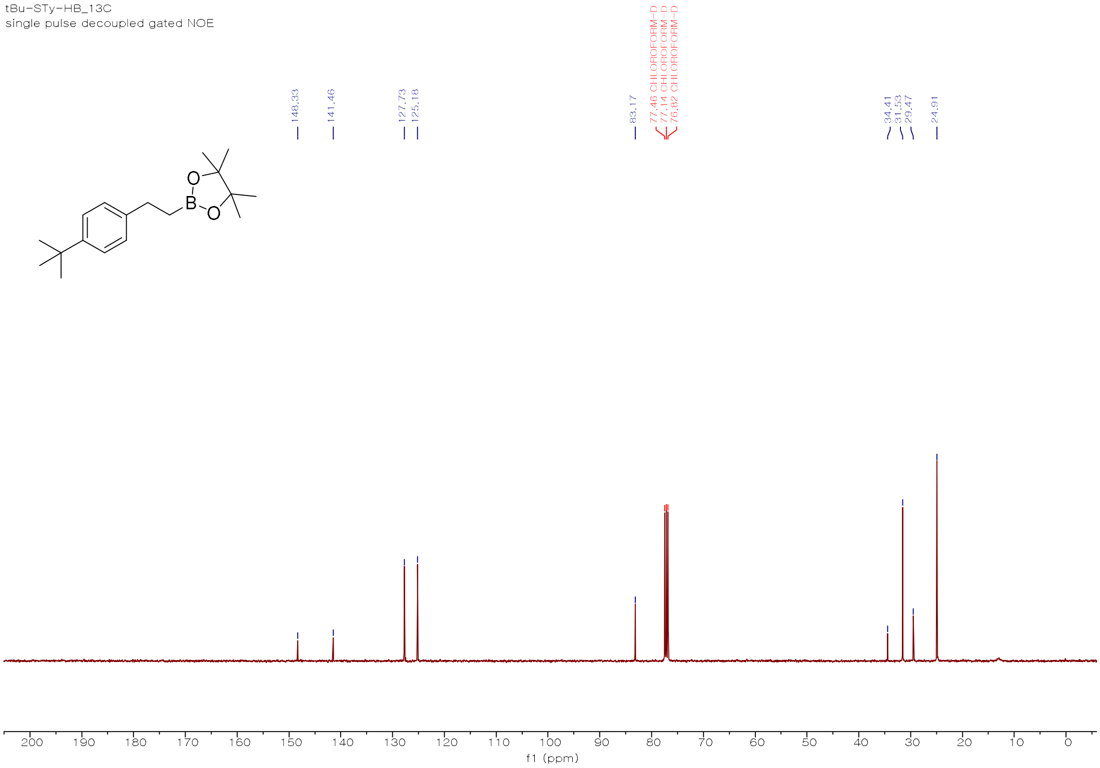

^{13}C NMR of 2-(4-(tert-butyl)phenethyl)-4,4,5,5-tetramethyl-1,3,2-dioxaborolane (**2g**)

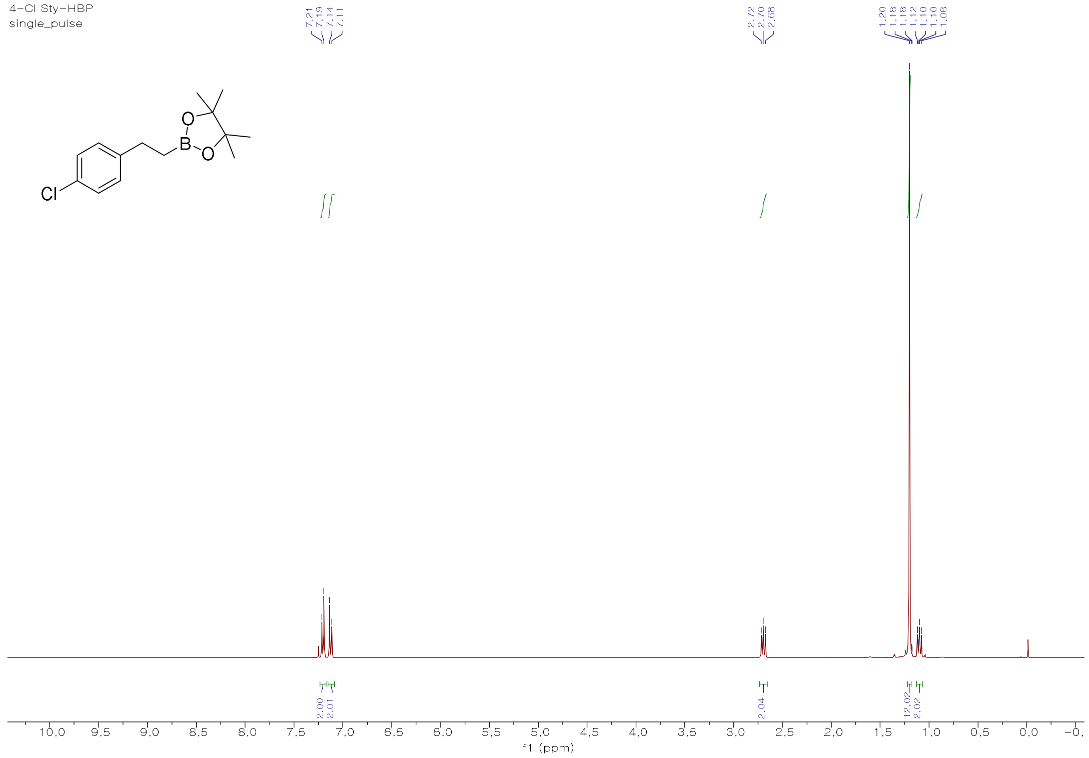

^{1}H NMR of 2-(4-chlorophenethyl)-4,4,5,5-tetramethyl-1,3,2-dioxaborolane (**2h**)

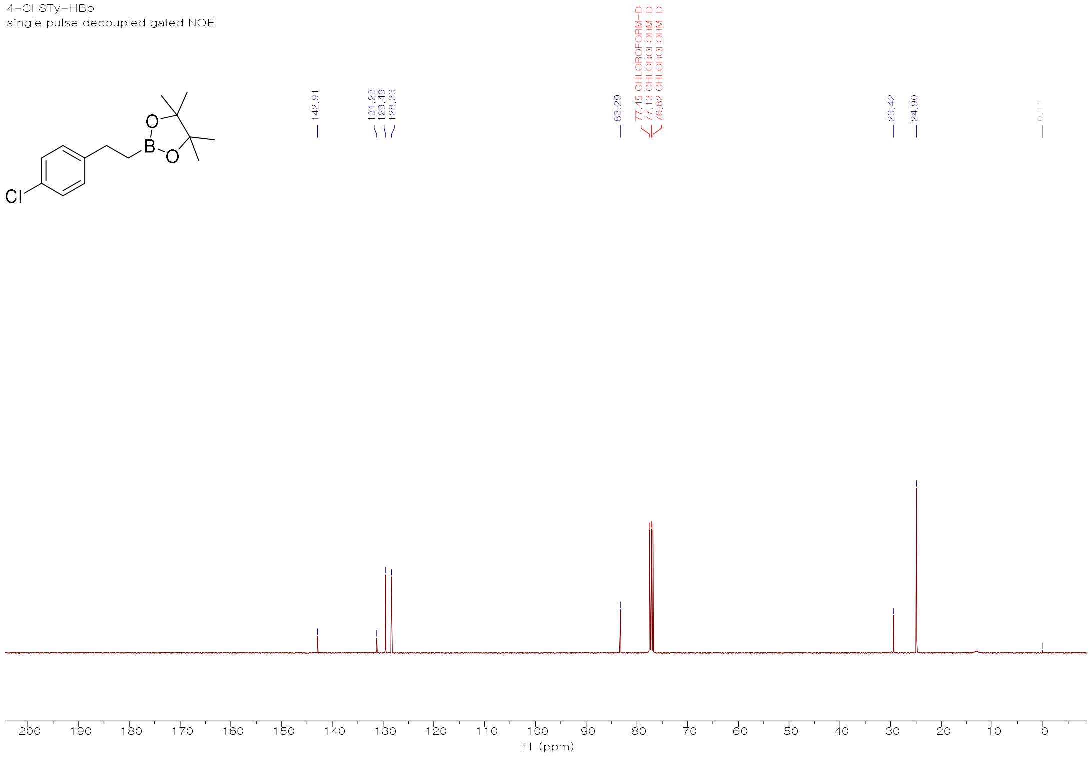

^{13}C NMR of 2-(4-chlorophenethyl)-4,4,5,5-tetramethyl-1,3,2-dioxaborolane (**2h**)

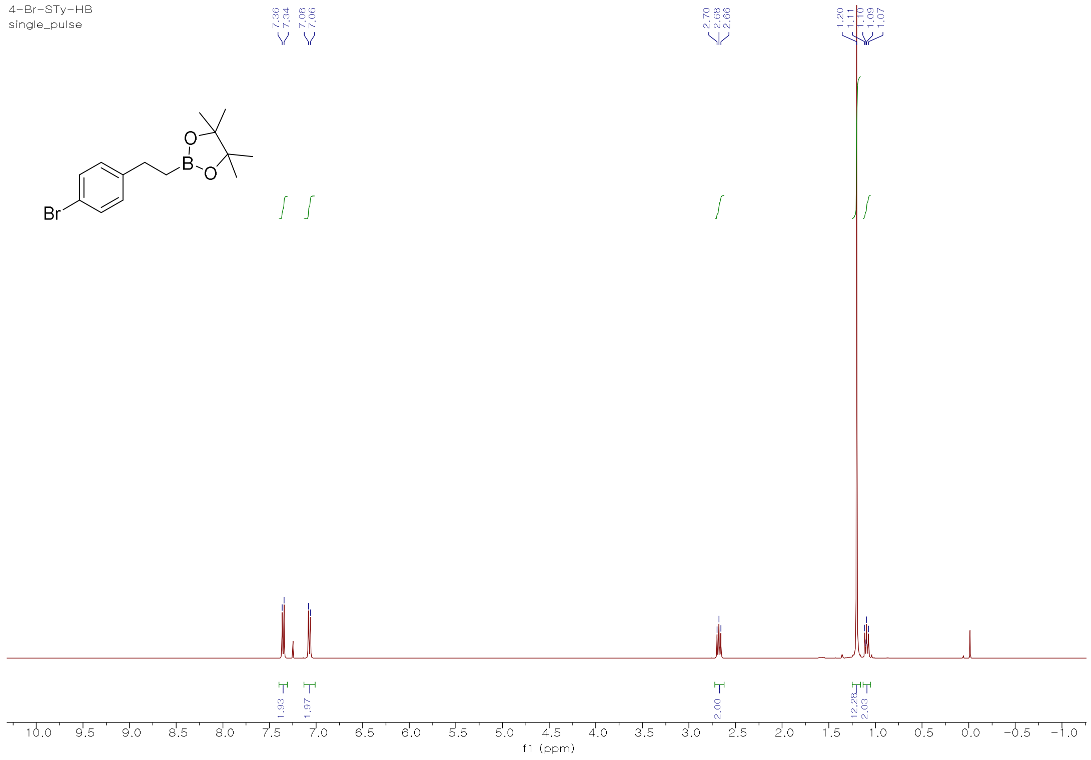

^{1}H NMR of 2-(4-bromophenethyl)-4,4,5,5-tetramethyl-1,3,2-dioxaborolane (**2i**)

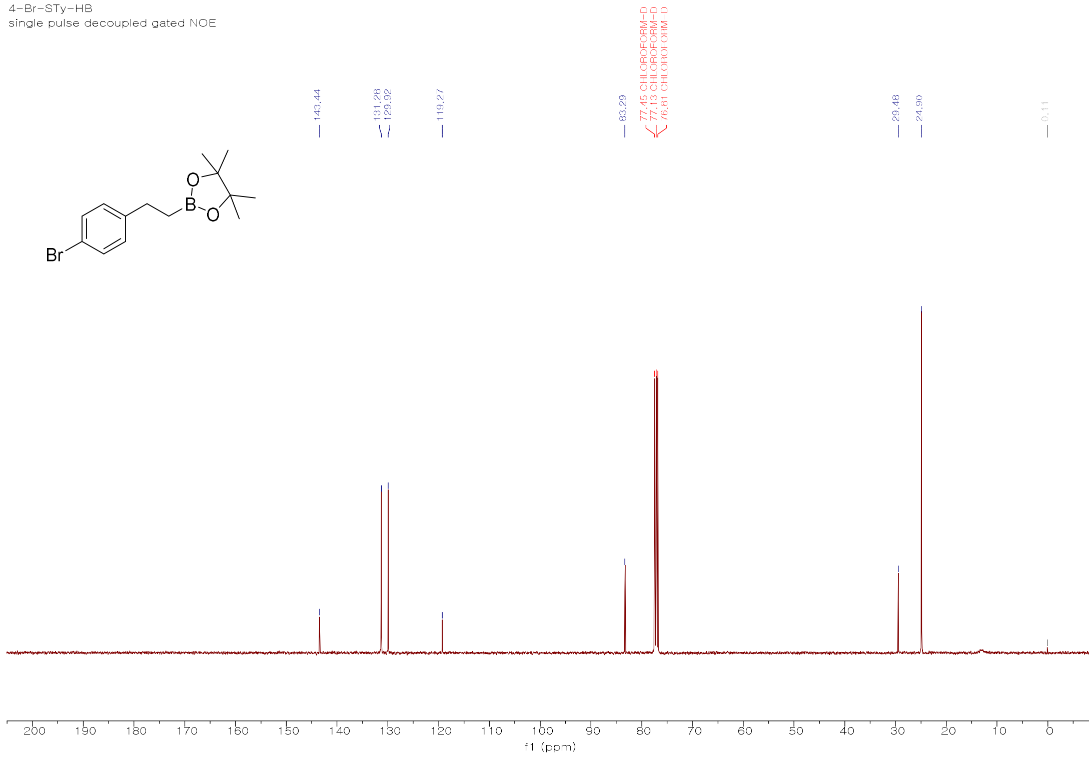

^{13}C NMR of 2-(4-bromophenethyl)-4,4,5,5-tetramethyl-1,3,2-dioxaborolane (**2i**)

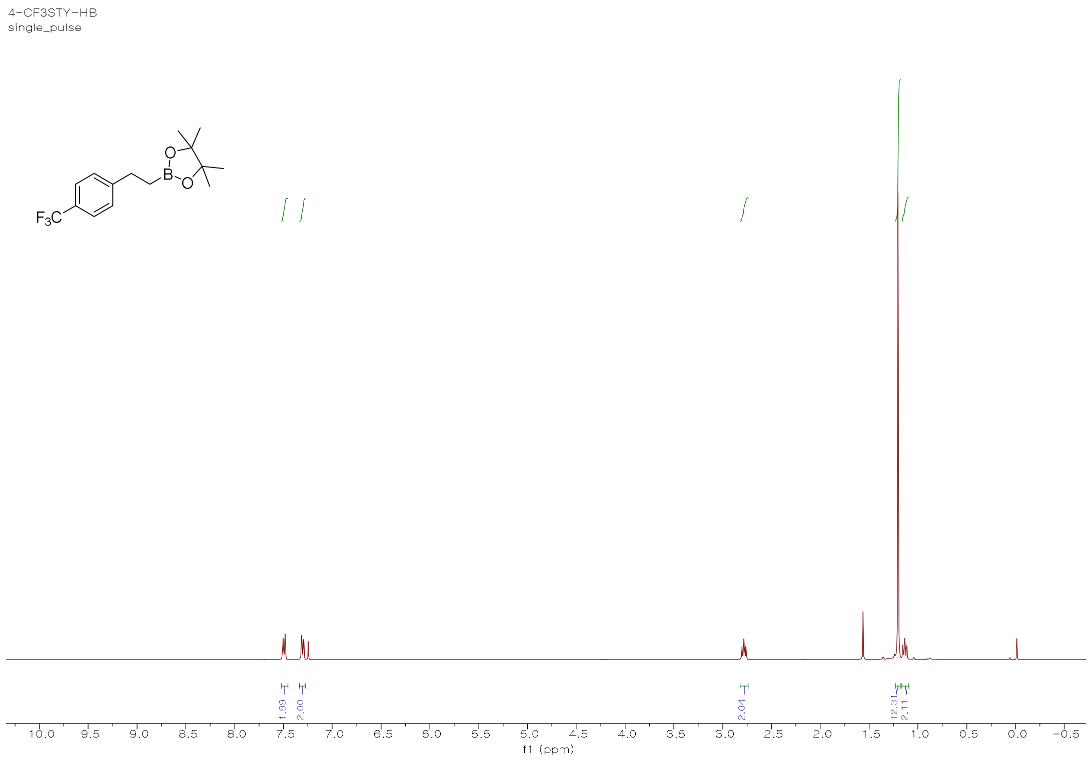

^{1}H NMR of 4,4,5,5-tetramethyl-2-(4-(trifluoromethyl)phenethyl)-1,3,2-dioxaborolane (**2j**)

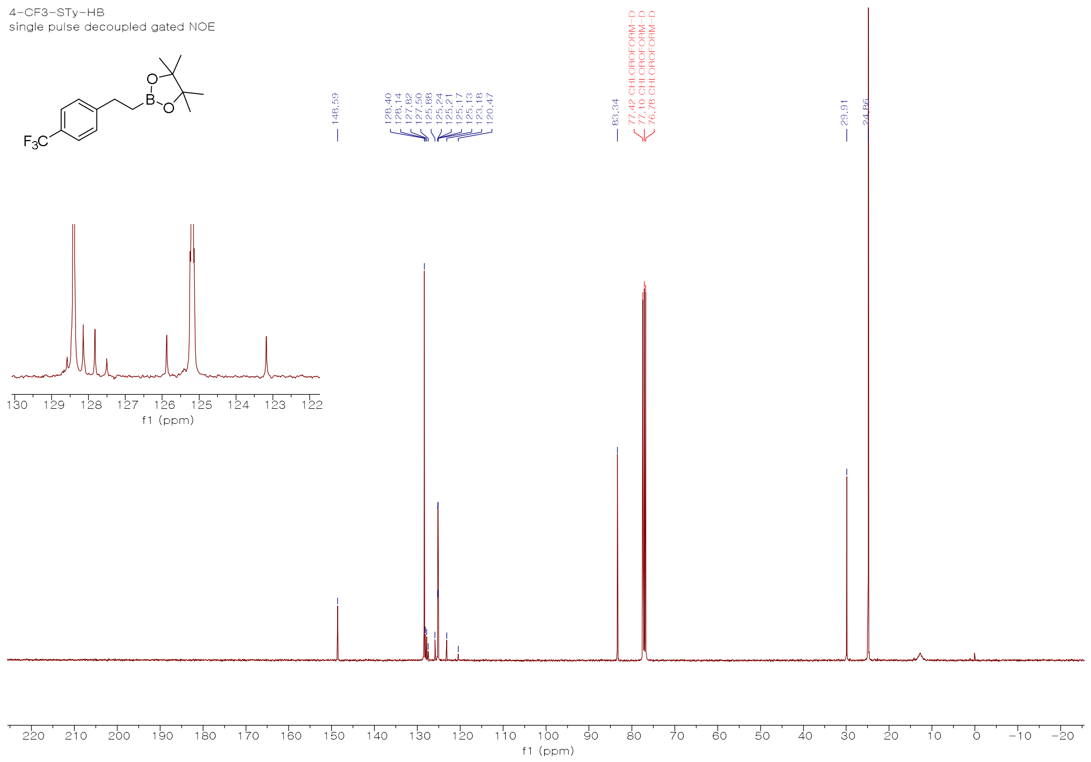

^{13}C NMR of 4,4,5,5-tetramethyl-2-(4-(trifluoromethyl)phenethyl)-1,3,2-dioxaborolane (**2j**)

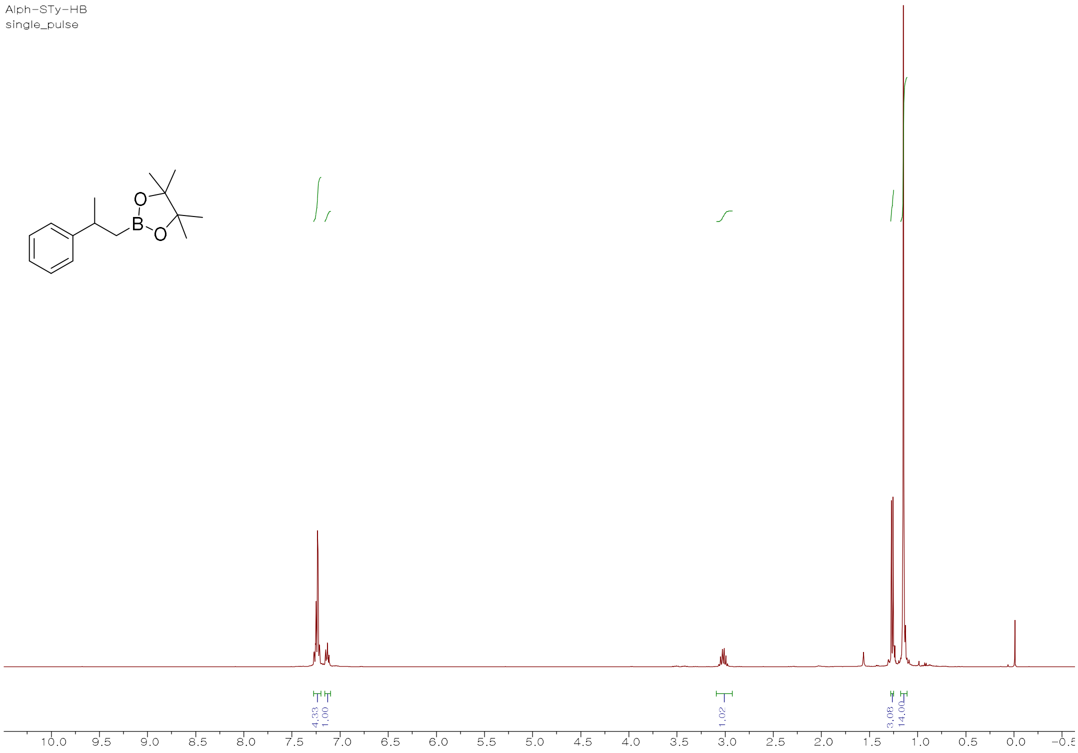

^{1}H NMR of 4,4,5,5-tetramethyl-2-(2-phenylpropyl)-1,3,2-dioxaborolane (**2k**)

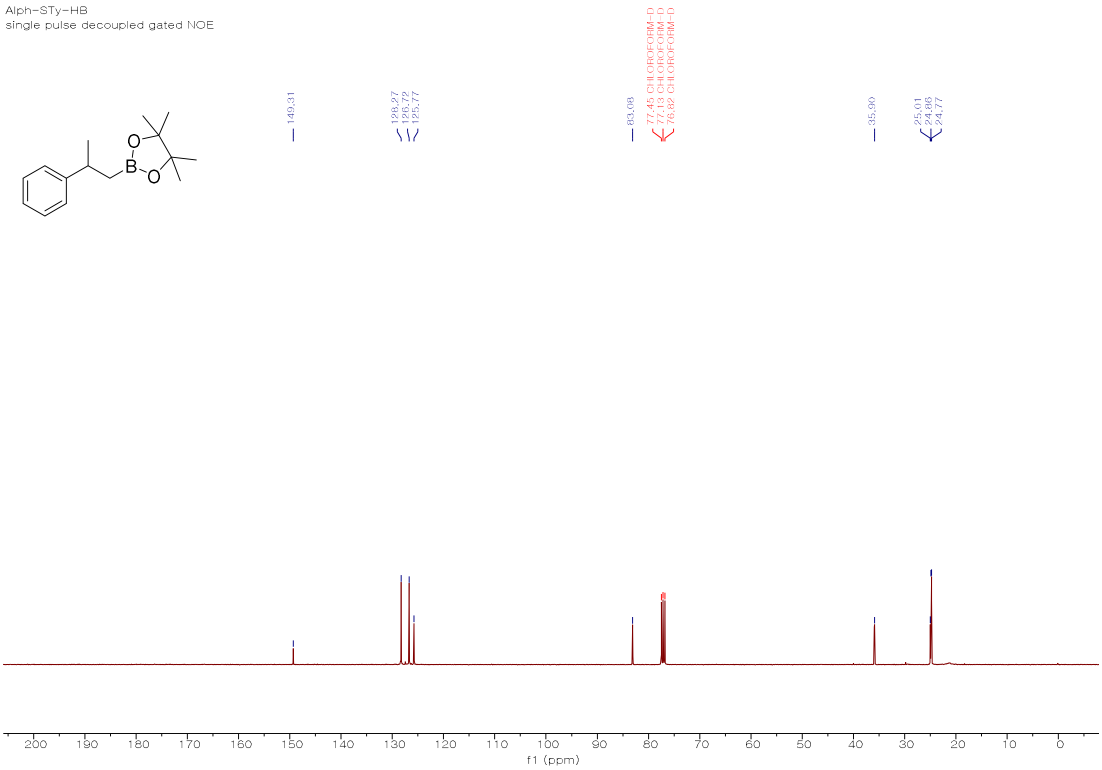

^{13}C NMR of 4,4,5,5-tetramethyl-2-(2-phenylpropyl)-1,3,2-dioxaborolane (**2k**)

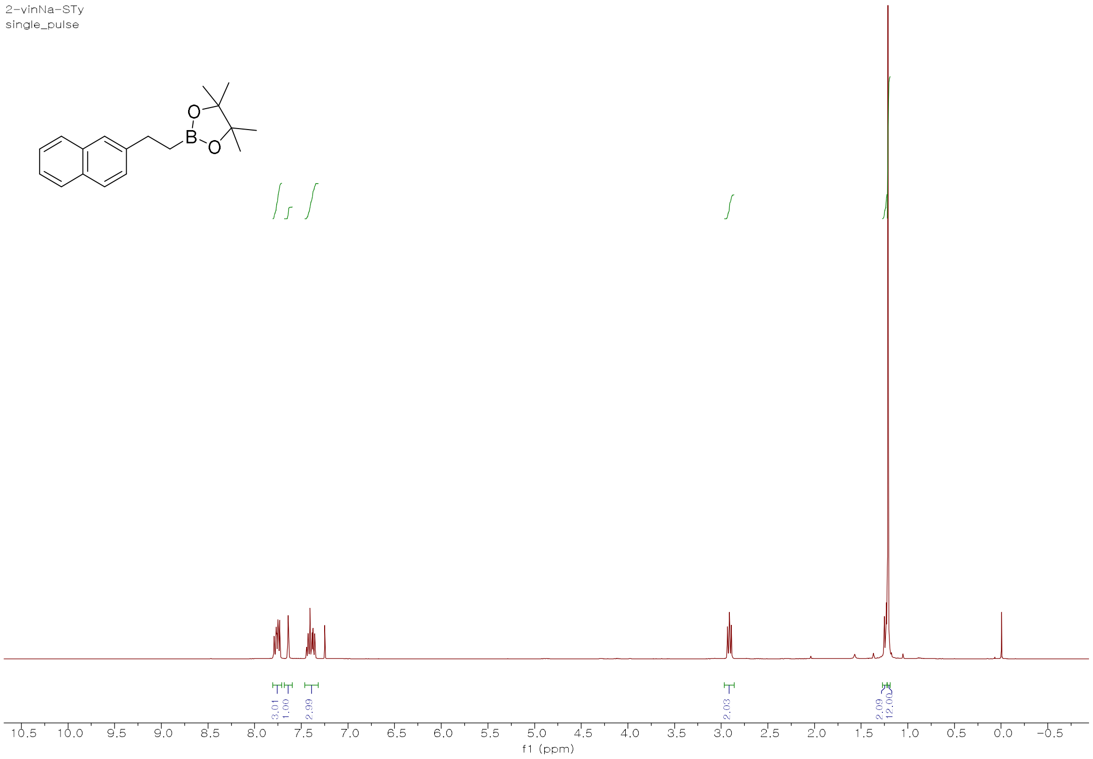

^{1}H NMR of 4,4,5,5-tetramethyl-2-(2-(naphthalen-2-yl)ethyl)-1,3,2-dioxaborolane (**2l**)

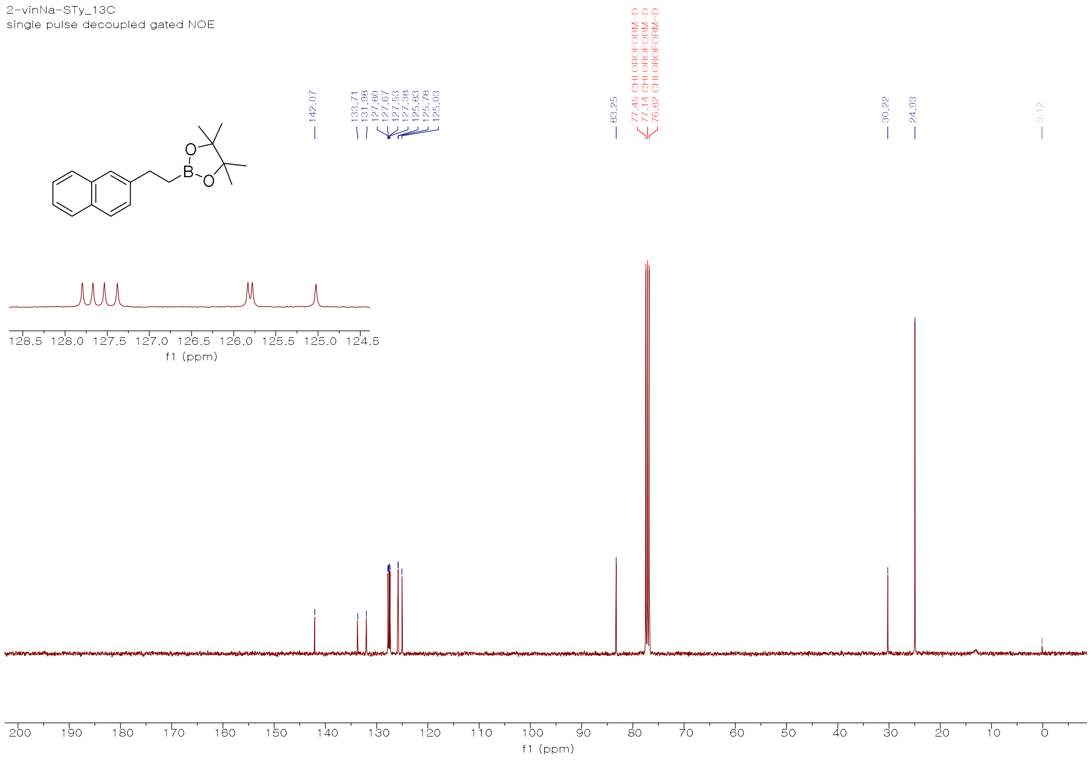

^{13}C NMR of 4,4,5,5-Tetramethyl-2-(2-(naphthalen-2-yl)ethyl)-1,3,2-dioxaborolane (**2l**)

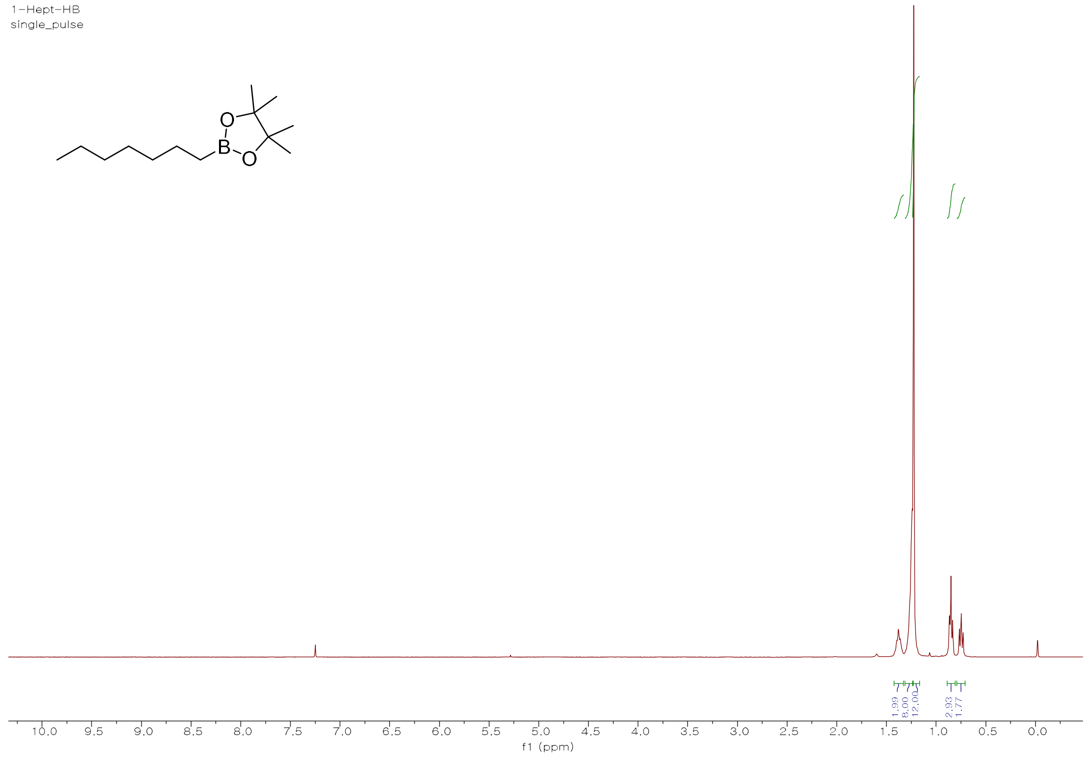

^{1}H NMR of 2-heptyl-4,4,5,5-tetramethyl-1,3,2-dioxaborolane (**2m**)

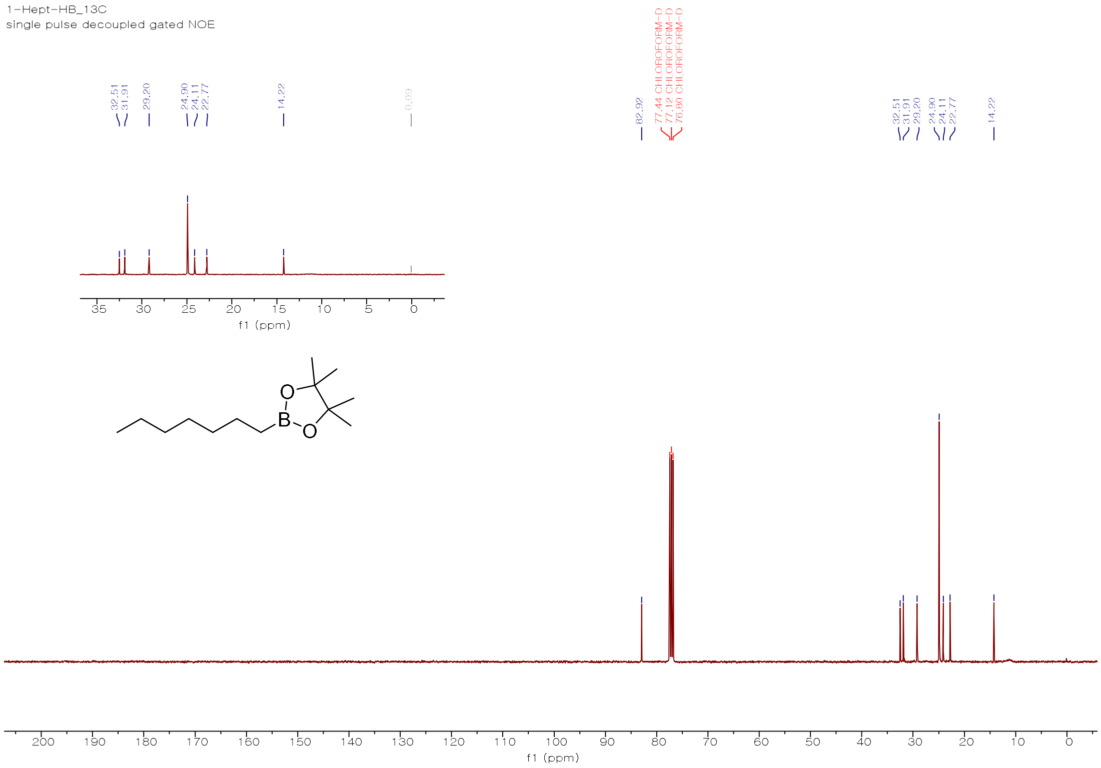

^{13}C NMR of 2-heptyl-4,4,5,5-tetramethyl-1,3,2-dioxaborolane (**2m**)

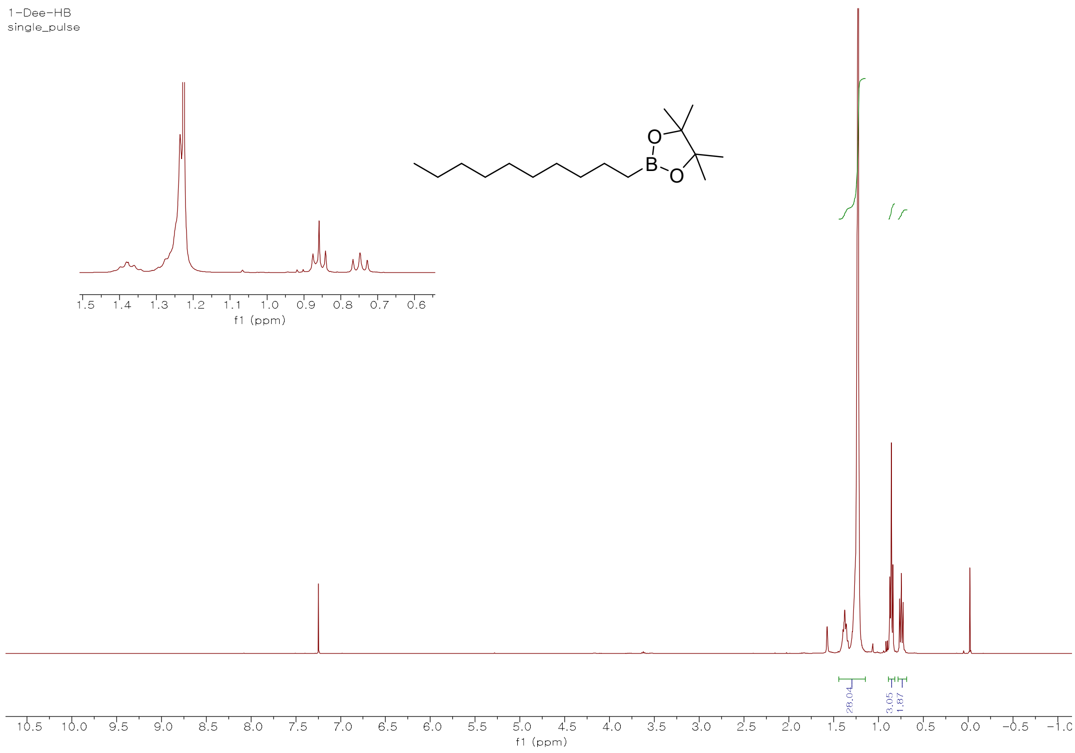

^{1}H NMR of 2-decyl-4,4,5,5-tetramethyl-1,3,2-dioxaborolane (**2n**)

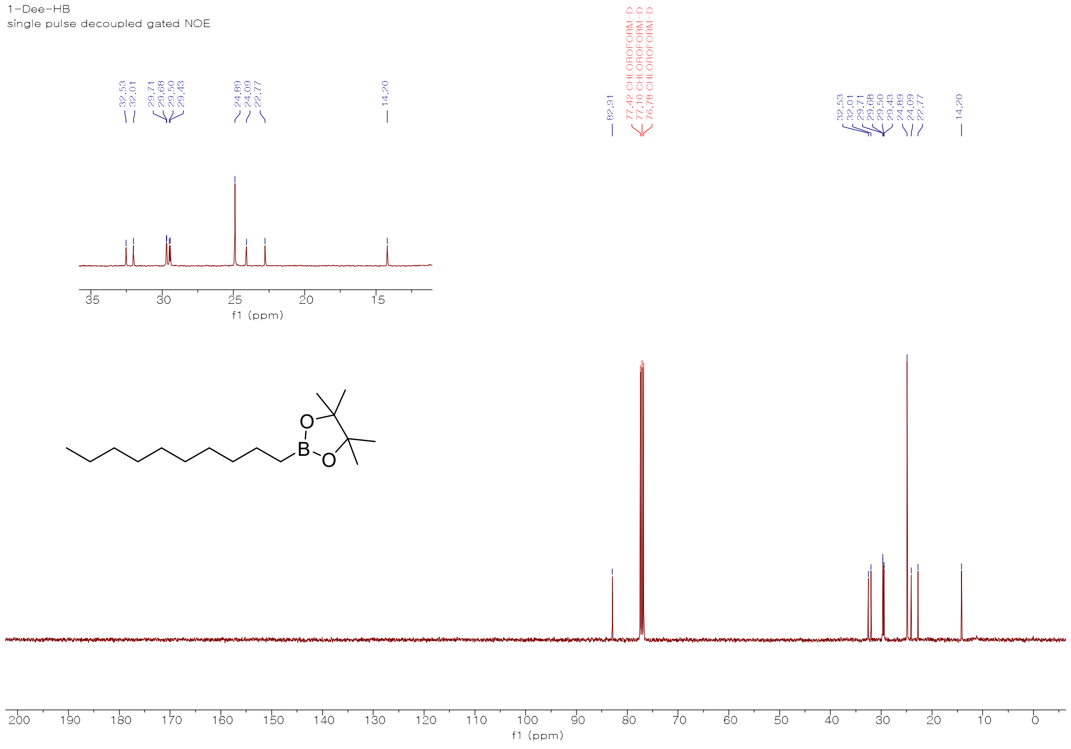

^{13}C NMR of 2-decyl-4,4,5,5-tetramethyl-1,3,2-dioxaborolane (**2n**)

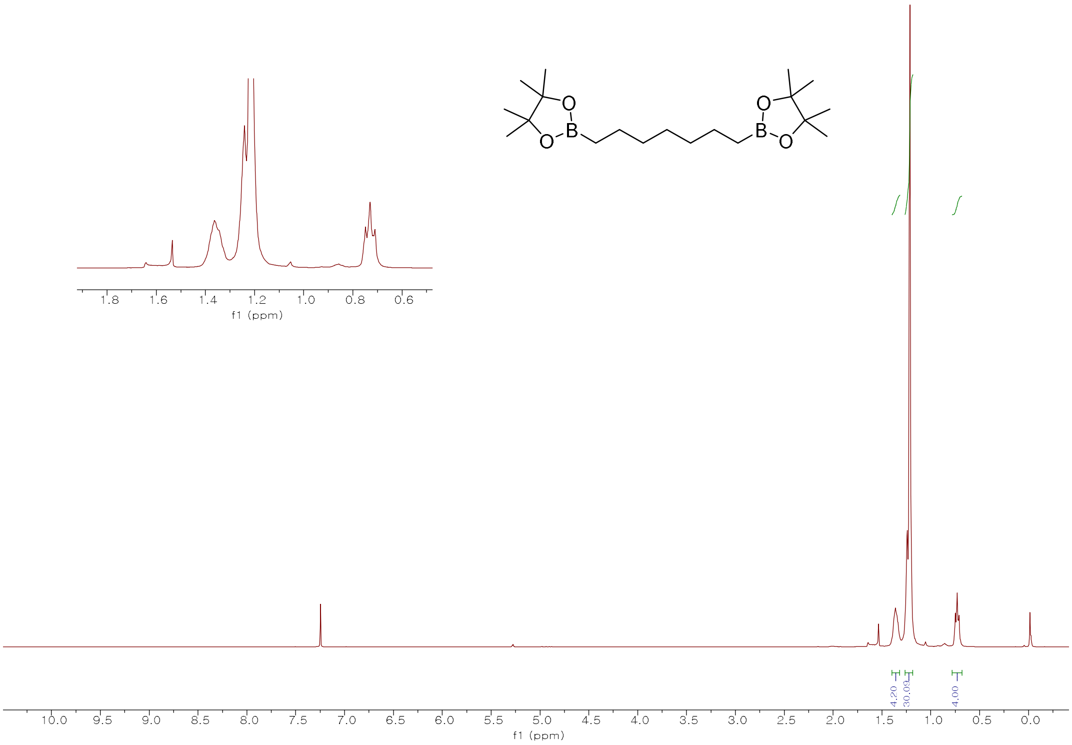

^{1}H NMR of 1,7-bis(4,4,5,5-tetramethyl-1,3,2-dioxaborolan-2-yl)heptane (**2o**)

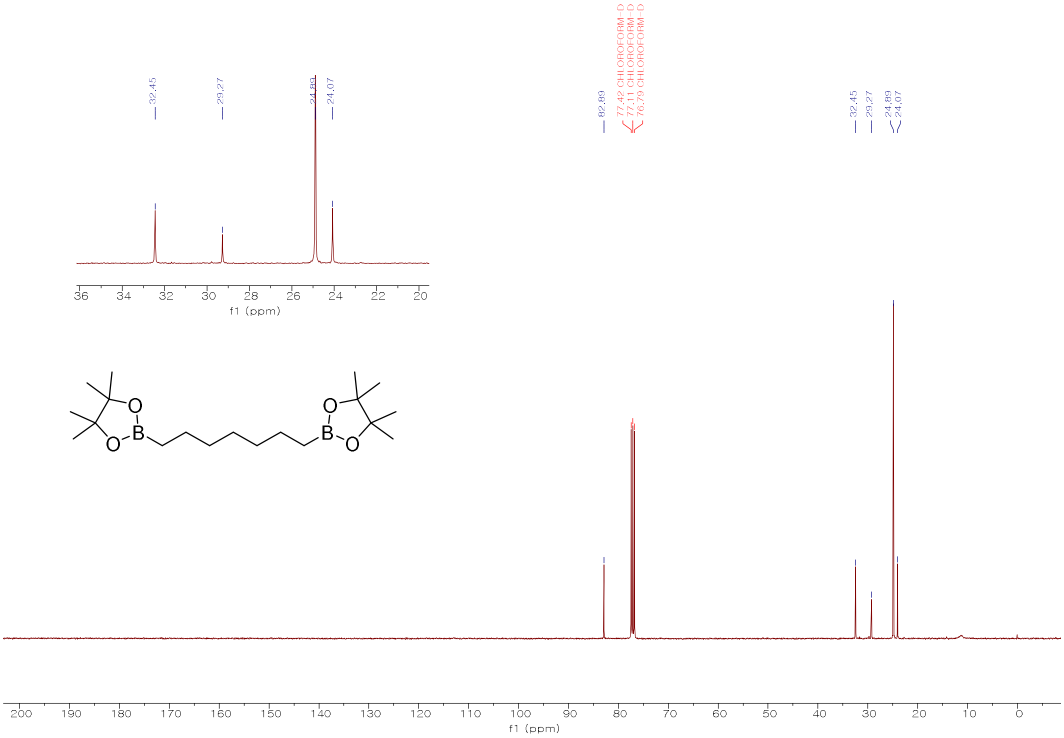

^{13}C NMR of 1,7-bis(4,4,5,5-tetramethyl-1,3,2-dioxaborolan-2-yl)heptane (**2o**)

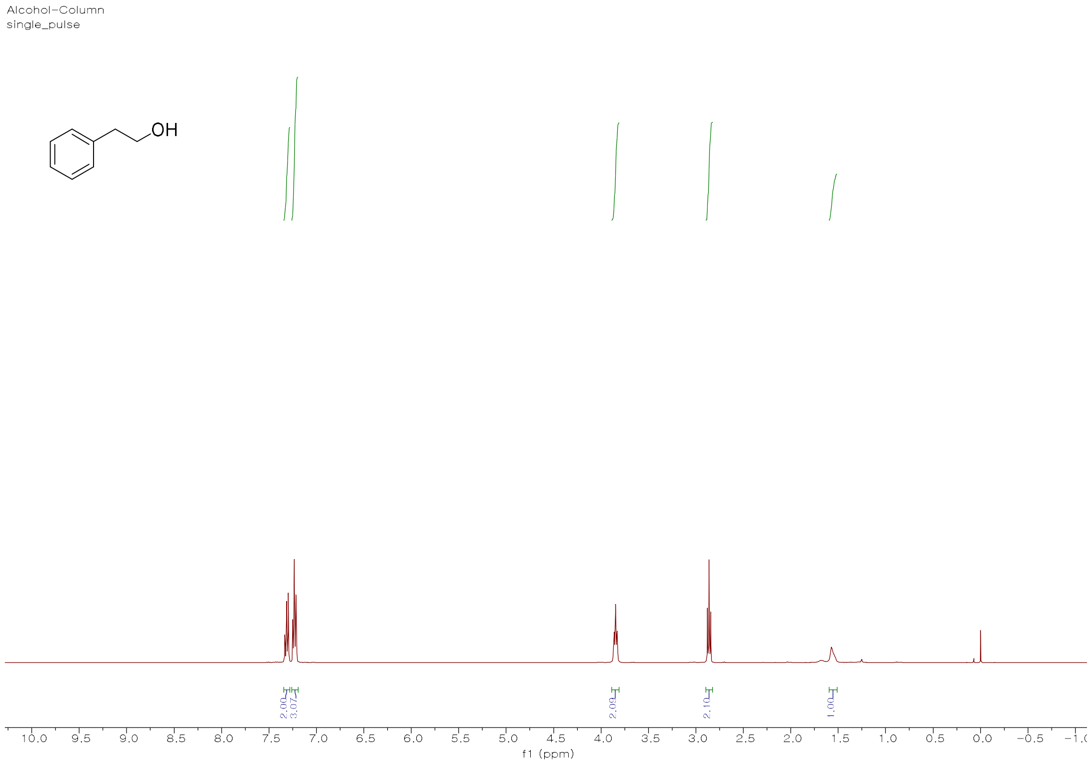

^{1}H NMR of 2-phenylethan-1-ol (**3a**)

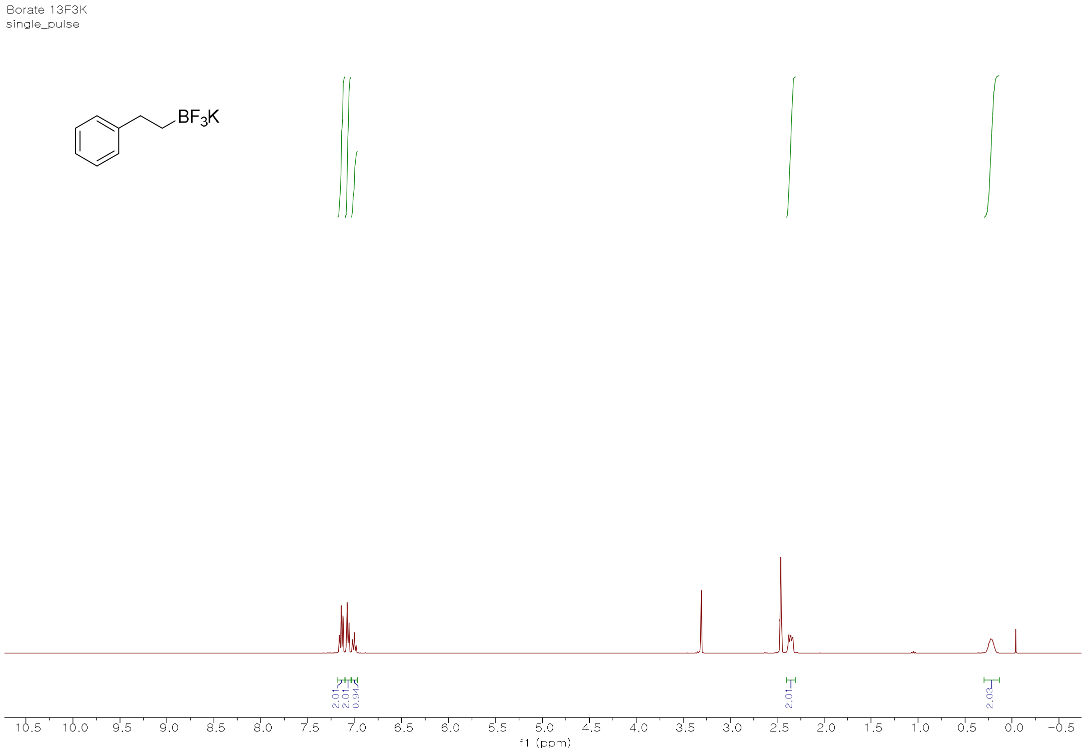

^{1}H NMR of potassium phenethyltrifluoroborate (**3b**)

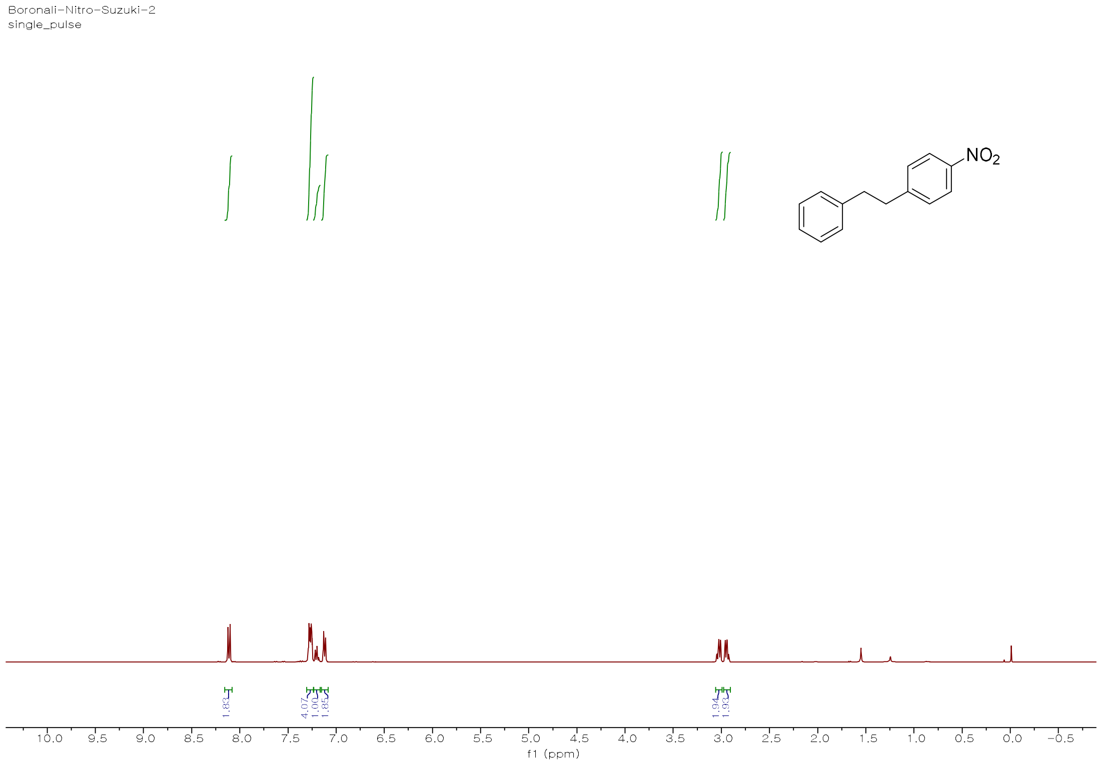

^{1}H NMR of 1-nitro-4-phenethylbenzene (**3c**)

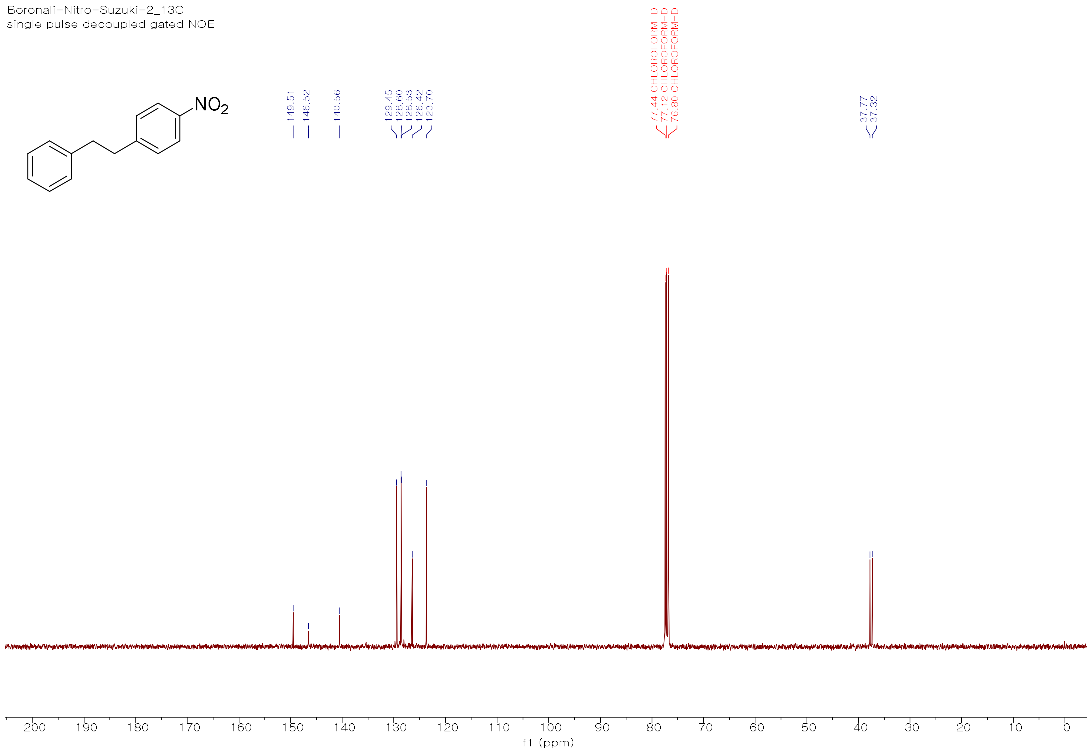

^{13}C NMR of 1-nitro-4-phenethylbenzene (**3c**)

4. References.

S1) K. Yanga, Q. Song, *Green Chem*., 2016, **18**, 932-936.

S2) L. Zhang, Z. Zuo, X. Leng, Z. Huang, *Angew. Chem. Int. Ed.* 2014, **53**, 2696-2700.

S3) (a) G. A. Molander, C. S. Yun, M. Ribagorda, B. Biolatto, *J. Org. Chem.* 2003*,* **68**, 5534-5539; (b) G. A. Molander, C. S. Yun, *Tetrahedron* 2002, **58**, 1465-1470.

S4) A. Bismuto, M.J. Cowley, S.P. Thomas, *ACS Catal*. 2018, **8**, 2001−2005.

S5) Q. Yin, S. Kemper, H.F.T. Klare, M. Oestreich, *Chem. Eur. J.* 2016, **22**, 13840-13844.

S6) G. Zhang, J. Wu, M. Wang, H. Zeng, J. Cheng, M.C. Neary, S. Zheng, *Eur. J. Org. Chem*. 2017, 5814–5818.

S7) S. Kisan, V. Krishnakumar, C. Gunanathan, *ACS Catal*. 2017, **7**, 5950−5954.

S8) P. Baran, C. Li, J. Wang, A. K. Chatterjee, M. Kumar, S, Yu, K. A. Johnson, T. Qin, M. Shang, PCT Int. Appl. (2018), WO 2018175173 A1 20180927.
